# Bioengineering methods for tailoring treatment of Osteomyelitis: Advancing strategies and application

**DOI:** 10.1016/j.mtbio.2025.102575

**Published:** 2025-11-19

**Authors:** Qi-Rui Geng, Ya-Xing Li, Heng Gong, Ting-Jiang Gan, Shi-Jiu Yin, Xi-Kun Ma, Cheng Zheng, Ye Wu, Hui Zhang, Yunbing Wang

**Affiliations:** a Department of Orthopedic Surgery and Orthopedic Research Institute, West China Hospital, Sichuan University, Chengdu, Sichuan, 610041, China; b National Engineering Research Center for Biomaterials, Sichuan University, Chengdu, Sichuan, 610065, China; c Med-X Center for Manufacturing, Sichuan University, Chengdu, Sichuan, 610041, China

**Keywords:** Osteomyelitis, Multifunctional biomaterials, Microenvironment regulation, Biological applications, Tissue engineering

## Abstract

Osteomyelitis (OM), an inflammatory disease of the bone due to pathogenic infection, represents one of the most significant challenges encountered by orthopedic surgeons worldwide. Under pathogenic, OM sites undergo persistent inflammation, oxidative stress, bone immune dysfunction, and abnormal osteocyte metabolism, resulting in recurrent bacterial infections and bone resorption. The curation of OM primarily relies on systematic high-dose antibiotics and local surgical debridement. Despite the discovery of various antimicrobial drugs in recent decades, there has been little clinical progress in OM eradication. The emergence of bioengineering materials offers great hope for addressing this issue. Beyond the antimicrobial activity, they often possess one or more of the following functions: antioxidant, anti-inflammatory, immunomodulatory, angiogenic and osteogenic. These materials have shown promising therapeutic effects in animal OM models. However, extensive and comprehensive animal studies and clinical trials are still needed to determine their direct and lasting impact on OM patients. This review integrates the complex microenvironmental characteristics of OM, delving into the construction principles and mechanisms of various bioengineering methods. It aims to enhance understanding of their suitability in meeting the therapeutic needs of OM. Additionally, it explores the clinical applicability and future prospects of multifunctional biomaterials in treating OM.

## Graphical abstract

Osteomyelitis treatment urgently needs a novel biomaterial to ease the current burdens.

**Figure:**
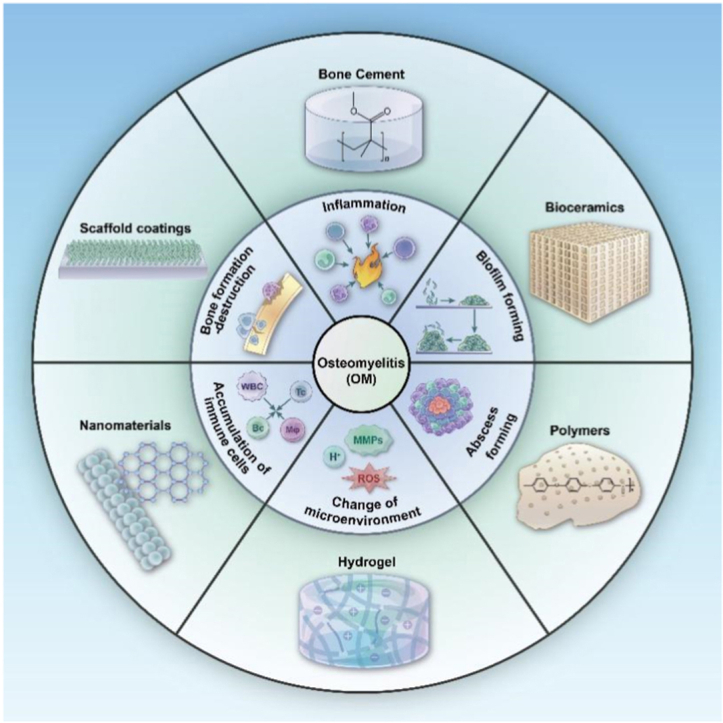

## 1 Introduction

Osteomyelitis (OM) is a typical orthopedic deep tissue infection, which is an inflammatory process often accompanied by bone destruction due to purulent microbial infections. The disease is often caused by the spreading of local trauma infection, bone surgery, or infections outside of the bone [1,2]. An epidemiological study showed that the incidence rate of OM is rising due to the increasing prevalence of risk factors, especially in diabetes and elderly. On average, 22 out of 100,000 people are infected with OM every year in the United States, with an average age of 52, and its incidence has been increasing in recent years [2]. During the curing process, patients may suffer from unbearable side effects of antibiotics and the painful process of multiple surgeries. Also, the cost of powerful antibiotics and surgeries is also a huge burden on patients, their families, and even the whole society. What is even worse is that if OM patients are not observed timely or carefully, they may have the risk of having to undergo amputation, which will lead to a significant drop of life quality.

Systematic use of antibiotics is the old-fashioned way for treating bacteria-induced OM. However, the abuse of antibiotics has caused the emerge of bacterial resistance, and can also lead to some unbearable side-effects caused by the increase of the dosage such as liver failure, kidney failure and hearing loss. Surgical debridement combined with local antibiotics and sustained-release systems has been proven to significantly improve the cure rate of chronic OM. However, surgical debridement is complex and can result in significant trauma and a high risk of postoperative infection [3,4]. In addition, the cost of surgical debridement is relatively high, which may impose a significant economic burden on patients. In recent years, the application of biomaterials has gradually gained the attention of scientists and become a popular research direction. The advantage of biomaterials is that it not only provides supporting to the impaired bone, but also act as a carrier, providing sustained release of drugs. The biological materials currently used to treat OM include polymethyl-methacrylate (PMMA) [5], bioceramics [6,7], sponges [8], hydrogels [9], and nanomaterials [10]. These materials can serve as good carriers for various antibacterial substances, achieve local treatments and reduce systemic drug concentrations, side effects caused by drugs, and the risk of drug resistance [[11], [12], [13]]. It is noteworthy that some of the biomaterials only serve as medicine carriers because of their low elastic modulus, such as hydrogels and nanomaterials. In addition, through modifications, the material can exhibit antibacterial activity for the treatment of OM [14]. More importantly, biomaterials can also exhibit various effects like antibacterial, antioxidant, and anti-inflammatory effects, and promote bone formation through chemical modification or by carrying drugs and active substances with different biological functions, further improving the treatment effect of OM [15,16].

At present, research on the use of biomaterials for the treatment of OM remains in its infancy, and most experiments are in the laboratory stage with limited clinical applications. This review introduces common biomaterials used for OM treatment, such as hydrogels, PEEK, and nanomaterials, and discusses the characteristics and construction principles of these materials. In addition, treatment strategies based on the aforementioned biomaterials are summarized, including different antibacterial strategies, multifunctional integration, such as anti-inflammatory/antioxidant/bone formation, and physically triggered antibacterial molecules. We discuss the current research status and progress of various functional bioactive materials for the treatment of OM in detail and their challenges in application prospects. This review may provide new ideas for the research and application of biomaterials to treat OM and provide new hope for the tailored treatment of OM in the future.

## 2 Pathological characteristics and common treatment methods for OM

### 2.1 Formation and pathological characteristics of OM

**Scheme 1:**
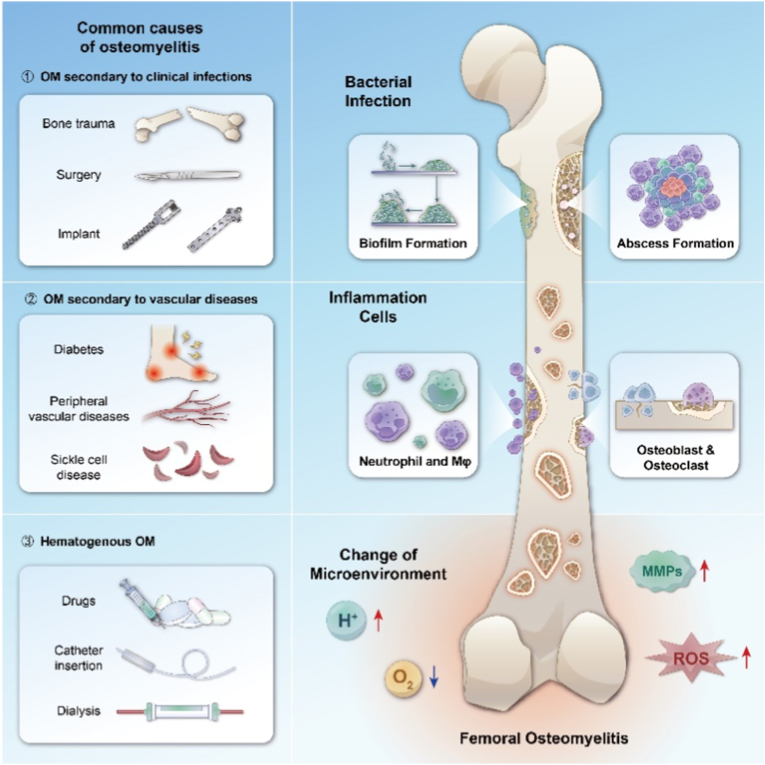
Common clinical types and pathological characteristics of OM Pictures on the left shows 3 common causes of OM: clinical infections, vascular diseases and hematogenous. The femur on the right summarizes the mechanics of OM: Bacterial Infection, Recruitment of inflammation cells, Imbalance of osteoblasts and osteoclasts, and Change of microenvironment. Biofilm formation and Abscess formation are two major causes of recurrent infection.

OM is often caused by the spreading of local traumatic infection, bone surgery, or joint replacement surgery. Pathogens can invade into the bone marrow through unsterilized wounds or surgical procedures. Common pathogens that cause OM include *Staphylococcus aureus (S.aureus)*, *Streptococcus hemolyticus (S.hemolyticus)*, *Pneumococcus*, *Escherichia coli (E.coli)*, *Pseudomonas aeruginosa (P.aeruginosa)*, and others. Among them, *S.aureus* is the most common pathogen of OM, accounting for approximately 75 % of the pathogenic spectrum of OM. OM can be divided into OM secondary to adjacent infection (orthopedic surgery, open trauma or joint implant infection), OM secondary to vascular disease (diabetes, peripheral vascular disease or sickle cell disease), and hematogenous OM (drug abuse, catheterization or kidney dialysis) according to the pathogenesis [1,17] (Scheme 1). Based on the duration of onset, OM can be further divided into the following subtypes: acute (onset within 10 days), subacute (onset within two weeks to one month), and chronic (onset after several months) [18]. Chronic OM is characterized by persistent infections lasting for months or even years, long-term inflammatory reactions, and the formation of fistulas and dead bones [1,19]. When this happens, it is even harder for the complete elimination of local bacteria, recurrent infection happens.

After pathogenic bacteria invade bone tissue, they grow and reproduce within and around the bone, releasing inflammatory factors and triggering inflammatory reactions. This marks the initiation of the interplay between bacterial infection and the host immune system: an excessive inflammatory response, while attempting to eradicate the pathogen, simultaneously begins to cause collateral damage to bone tissue. These activities lead to the destruction of bone tissue and surrounding tissues and the spread of pathogenic microorganisms [1]. The formation of biofilm plays an indispensable role in OM infection [20]. Bacteria with small colony variants (SCVs) such as SCV-*S.aureus* can form stable tiny colonies on the surface of implants or wounds [21], creating a quorum-sensing regulatory system for various bacteria within the membrane [2], which greatly increases the resistance of bacterial populations to adverse factors and their metabolic diversity. The head-scratching recurrent infection is mostly induced by the infection of SCVs. The formation of abscesses is also an important mechanism by which *S.aureus* acquires strong resistance [22]. The coagulase on the surface of *S.aureus* can form relatively loose fibrin on its surface, forming preliminary protection. This mechanism also contributes to recurrent infection of OM. In addition, various other immune cells play their positive or negative role, such as neutrophils and macrophages. Change of the microenvironment surrounding the inflammation site is an important cut-in point in the current researches, including low pH value, high level of reactive oxygen species (ROS) and local rising *matrix metalloproteinases* (MMPs). Anaerobic metabolism produces a large amount of lactic acid, vascular damage, poor blood circulation, and the inability to quickly transport acidic products further reduce the local pH value of the inflammatory microenvironment [23]. ROS are produced by various bactericidal enzymes during the process of killing pathogens, mainly include O^2−^, H_2_O_2_, hydroxyl radicals, etc. [24] MMPs are secreted by immune cells participating the immune process, regulating the process of tissue regeneration locally. Local inflammation includes tissue damage and obstruction of the blood supply capacity of blood vessels. The aforementioned alterations in the microenvironment represent a manifestation of the vicious cycle of "bacterial infection-immune response-tissue damage." At the molecular level, this interplay is driven by specific pathways: Bacterial Pathogen Associated Molecular Patterns (PAMPs) (e.g., LPS) binding to immune cell Pattern Recognition Receptors (PRRs) (e.g., TLR4) activate key signaling pathways like NF-κB, triggering a cascade of pro-inflammatory cytokines (TNF-α, IL-1β). This recruits and activates neutrophils, which release ROS and proteases (e.g., neutrophil elastase) that, while intended for pathogens, directly degrade extracellular matrix and damage host cells. The activated inflammasome cascades further amplify inflammation through IL-1β maturation and pyroptosis. Simultaneously, a dysregulated repair phase is characterized by the upregulation of immunosuppressive molecules like TGF-β and PD-1, which inhibit effector T-cell function and promote fibrotic responses, ultimately facilitating bacterial persistence.

During the process of inflammation, precursor immune cells gather and make the initial step of killing bacteria to release antigen. Then, T cells and B cells recognize the antigen and do their work respectively. Cytotoxic T cells lead to dysfunction, inhibits the secretion of killing substances (e.g. perforin, serine esterase and interferon), which downregulate the elimination of local bacteria. At the same time, regulatory T cells also upregulates the secretion of inhibitory substances (e.g., TGF-β,IL-4 and IL-10), which further inhibit other immune cells like cytotoxic T cells to do their work in bacteria elimination [25]. B cells mediate cellular phagocytosis by secreting antibodies, but cannot act as a critical role. Imbalance of bone destruction and reconstruction are caused by the imbalance of M1 and M2 macrophages induced by pathogens [26]. They cause the imbalance indirectly by regulating the activity of osteoblasts and osteoclasts. Thus, a clear picture emerges of how "the interplay between bacterial infection, the immune system, and tissue damage" forms a self-reinforcing feedback loop: pathogens persist and disrupt immune recognition through strategies like biofilms and SCVs; in response, the dysregulated immune response (e.g., impaired cytotoxic T cells and abundant secretion of inhibitory substances) not only fails to clear the infection but also exacerbates tissue inflammation and destruction; the resulting damaged tissue and hypoxic, acidic microenvironment further cripple immune cell function, providing a niche for persistent bacterial survival. According to the mechanisms mentioned above, the clinical features of OM include stability of microorganisms, recurrent infection, difficulty curing and prolonged healing process. So, tailoring the function of eliminating microorganisms, regulating local microenvironment, promoting angiogenesis and immunoregulating should be made into consideration when constructing biomaterials.

Therefore, the treatment for OM mainly includes the following steps: i) clearing the source of infection; ii) preventing secondary infections; iii) achieving anti-inflammatory and antioxidant affects, as well as constructing and maintaining a stable internal environment that promotes bone formation; and iv) performing implantation, which is inevitable when biomaterials are used to treat OM and involves incorporating the implant into the bone function.

### 2.2 Diagnosis and treatment

Typical acute OM has a series of untypical syndromes such as fever, pain, warmth, redness and swelling of local infection site, lethargy, etc. Chronic OM adds drainage from the open wound from the infected bone, sometimes includes local tenderness. Fever only happens in some of the cases. The diagnosis of OM requires a comprehensive consideration of medical history, physical examination, and laboratory examination. Diagnoses commonly include the following features: i). fever, sinus, local pain, and other physical symptoms; ii). Blood tests, such as tests on white blood cells, CRP, and ESR; iii). X-ray, CT, MRI, bone scan, and other imaging examinations; and iv). Bone tissue biopsy or postoperative pathological examination [1].

The common treatment methods for OM mainly include appropriate antibiotic application and surgical treatment. The use of high-dose antibiotics throughout the body was once the main method of treating OM, and various antibiotics have been used in the treatment. It was widely used due to the simple and practicable use and the high efficacy of bactericidal effect. In order to reduce the concentration of antibiotics throughout the body, local application of antibiotics is becoming an emerging research direction. The main method is to use carriers to carry and deliver antibiotics directly to the inflammatory site through injection or implantation methods so that the local antibiotics reach the antibacterial concentration and maintain a certain period, achieving the effect of local sterilization. It can lower the systematic concentration of antibiotics, reducing side effects. Surgical treatment of OM involves thorough debridement to achieve infection control. This method achieves a certain therapeutic effect on chronic OM with poor drug treatment [27].

However, various shortcomings still restrict the widespread use of these methods. The infection site of OM has few blood vessels, poor blood circulation, and local biofilm formation [28], making it difficult for antibiotics to reach antibacterial concentrations in inflamed areas. In addition, the abuse of antibiotics has led to a sharp increase in bacterial resistance and a decline in the efficacy of traditional antibiotics [29]. The therapeutic effect of applying large doses of antibiotics throughout the body is poorer than before. Moreover, the side effects of high-dose antibiotics on the human body cannot be ignored. About the local use of antibiotics, many issues need to be addressed. For example, infection issues during the implantation process, quality of life issues for patients after implantation of the carrier, and whether the carrier needs to be removed through a second surgery after rehabilitation. Surgical treatment also meets Its drawbacks. First, surgically removing a large amount of bone and soft tissue can cause great harm to the human body and seriously affect the patient's quality of life. The pain of patients during surgery and rehabilitation is also severe. Second, surgical debridement cannot guarantee the full elimination of potential infections, and if antibiotic treatment is not continued, the recurrence rate is very high. Moreover, the risk of secondary infection during surgery cannot be avoided.

Using biomaterials as carriers to put drugs directly to the infection site is becoming a hot point in researchers. They focus on how to utilize biomaterials to avoid the drawbacks of traditional carriers. Biomaterials are stars in the medical field due to their relatively good biocompatibility, extremely high plasticity, and unique properties; due to these properties, biomaterials can combine multiple substances. Currently, researchers have made new biomaterials that can carry many kinds of bactericidal agents to the infection site [30]. Other kinds of biomaterials have also emerged in other aspects.

## 3 Commonly used biomaterials in OM treatment

**Scheme 2:**
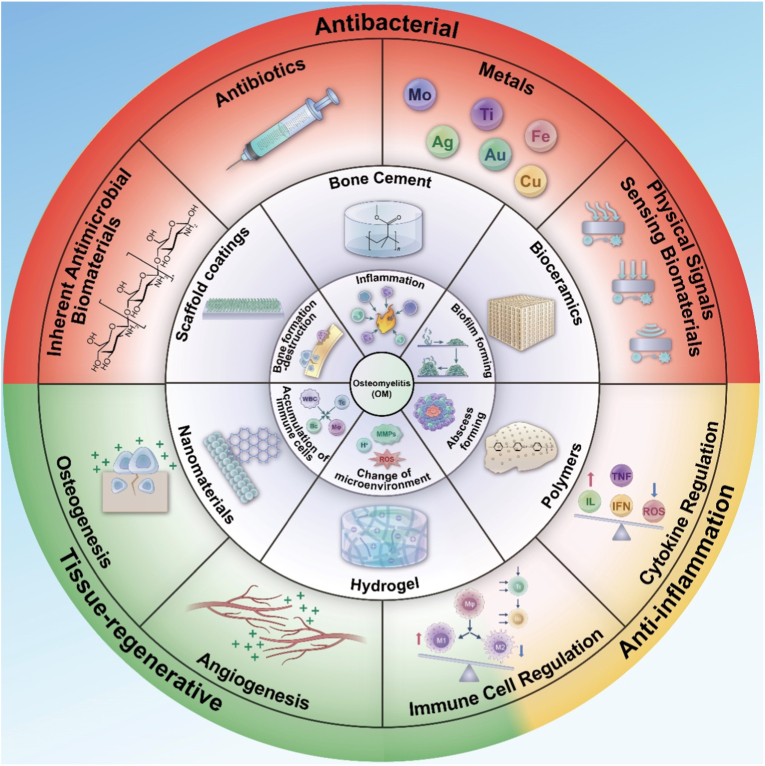
Brief introduction of OM, biomaterials and mechanisms of biomaterials treating OM. Center shows “Osteomyelitis (OM)”. The inner ring summarizes 6 main mechanisms of OM. Middle ring summarizes the 6 widely used biomaterials used to cure OM. Outer ring shows the mainly utilized mechanisms of biomaterials to cure OM. The outmost ring along with red, yellow and green colors shows 3 different ways to cure OM, corresponding with the mechanisms described in the outer ring.

Biomaterials are a class of natural or synthesized functional materials that are used to contact and interact with living systems and are capable of diagnosing and treating their cells, tissues and organs, replacing and repairing them or inducing regeneration. The use of biomaterials to treat OM is a manifestation of the application of biomaterials in medicine. Currently, biomaterials are having a wider use in clinical medicine. The biomaterial used to treat OM must tolerate the complex environment in the body without infection or damage. In response to the characteristics above, the biomaterials need to possess the following characteristics: i) excellent antibacterial ability; ii) good biocompatibility; iii) anti-inflammatory and antioxidant activities; iv) elastic modulus matching with bone tissue; v) the ability to promote bone tissue regeneration and repair; vi) certain biodegradability. Scheme 2 below is an introduction to some common materials used to treat OM.

### 3.1 Bone cement

**Fig. 1:**
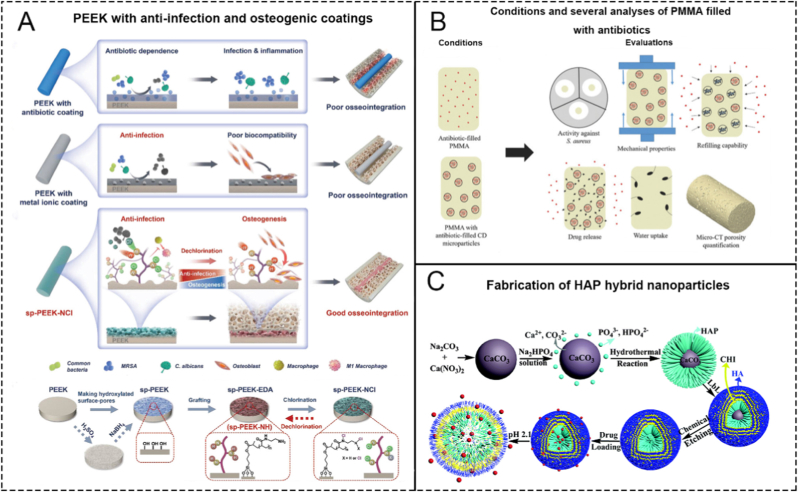
Schematic illustrations of the utilization of PEEK bone cement and HAp bioceramics. A. Schematics showing the surface coating on PEEK bone cement to enhance the osseointegration ability. Reproduced with permission from Ref. [46]. B. Graphics of the analyses carried out on PMMA bone cements. Reproduced with permission from Ref. [42]. C. The fabrication of HAP hybrid microparticles. Reproduced with permission from Ref. [47].

Bone cement is a commonly used biomaterial in orthopedic surgery. It gets its name from its appearance and some physical properties that are similar to common cement. Due to its excellent malleability, it is widely used in orthopedic implant surgeries and has also gained favor in the prevention and treatment of orthopedic infections [31]. Currently, there are four types of bone cement that have matured and are commercially applied: Polymethylmethacrylate (PMMA), Calcium Phosphate (CPC), Calcium Sulfate (CSC), and Magnesium Phosphate (MPC) bone cement [32]. In the field of OM treatment, researches utilizing the aforementioned bone cements to deliver antimicrobial, osteogenic, and wound healing drugs continue to emerge [33,34]. The majority of studies involve using PMMA to deliver antibiotics for the treatment of OM (Fig. 1B). While there are also studies on the use of other types of bone cement, the number of publications is relatively smaller. Here, we will focus on PMMA bone cement to introduce the application of bone cement in the treatment of OM.

Polymethylmethacrylate (PMMA) bone cement is the gold standard biomaterial for local antibiotic therapy in orthopedics and has been used for over 35 years for prophylaxis and therapy. In the 1970s, researchers used bone cement loaded with antibiotics to prevent or treat bone infections and achieved good clinical efficacy [35,36]. PMMA bone cements are easily available and can be made to contain either aminoglycosides (tobramycin and gentamicin) or glycopeptides (vancomycin), which are often used to prevent infection after arthroplasty and two-stage arthroplasty revision procedures [37,38]. However, PMMA bone cement is often not used to treat OM for the following reasons: (i) The choice of antibiotic concentration is controversial, low-dose antibiotics may lead to the formation of drug-resistant bacteria [39], and high-dose antibiotics adversely affect the physical properties of materials [40,41]. At present, the choice of the dose is usually decided by the surgeon in charge, lacking a unified standard of treatment [42]. (ii) Surgeons often face limitations in regard to selecting suitable antibiotics that can be safely mixed into PMMA, and compatibility issues arise with certain antibiotics, as some are heat-labile and unable to withstand the exothermic polymerization reaction of PMMA, while others, such as rifampin, hinder PMMA polymerization by scavenging free radicals. (iii) Another significant drawback of PMMA is its nonbiodegradability. Due to this property, PMMA must be removed after an infection is managed to prevent the hindrance of bone defect healing [43].

In general, commercial antibiotic-loaded PMMA bone cement is effective for preventive purposes but may not be suitable for treating established OM. Several studies have explored the addition of antibiotics with enhanced potency to PMMA bone cement. For instance, linezolid has been incorporated into PMMA and shown to prevent the spread and split of methicillin-resistant *S. aureus* [44]. In another approach, rifampicin, known for its high effectiveness against *S. aureus*, was encapsulated into alginate microcapsules before being added to bone cement. This method resulted in improved antibacterial activity, increased release of rifampicin compared to free drug, and maintenance of the mechanical properties of the cement [45]. However, these approaches still have limitations in the treatment of chronic or recurrent infections, as the approaches cannot be replaced with antibiotics after implantation. Therefore, the current development and utilization of antibiotic-loaded PMMA for treating OM primarily focuses on enhancing the material's antimicrobial properties, prolonging the release period of antibiotics, and increasing the degradability of bone cement materials. These approaches can promote bone regeneration after infection control, prevent secondary infections, and inhibit the formation of drug-resistant bacteria.

### 3.2 Bioceramics

Inorganic bioceramics have been well studied for bone regeneration due to their similarity with bone minerals. In addition to being biodegradable and osteocompatible, inorganic bioceramics showed improved osteoconducting activity associated with the formation and deposition of calcium [48]. Since 1892, ceramics have been used in the manufacturing of orthopedic implants. However, at that time, the application only used ceramics as inert materials to provide support, without considering the issue of interaction with the biological environment in the body; these ceramics were called the first generation of bioceramics. Currently, most implants modify ceramics, making their surface similar to the minerals of the bone, to obtain a stable binding interface with the bone [49,50]. Modified bioceramics can perform various functions, such as promoting bone formation and strengthening bone connectivity, which is the direction of development for second- and third-generation bioceramics today. These materials focus more on interaction and integration with the biological environment within the body.

Hydroxyapatite (HAP), as new-generation bioceramics, has become among the shiniest rising stars of bone repair materials due to its significant osteoconductivity, induction of bones, and reabsorption ability in vivo. Due to these properties, HAP can be widely applied in the treatment of orthopedic diseases. HAP can also be loaded with various antibacterial agents and can eliminate most of the drawbacks of PMMA bone cement [51]. HAP has been applied in bone fillers and tissue engineering scaffolds, as well as surface coatings for general implant materials; in addition, HAP can serve as a carrier for drug delivery. In the treatment of OM, many studies have used HAP loaded with anti-inflammatory drugs, antibacterial drugs, and osteogenic drugs, and these materials are already relatively mature. Many studies have focused on HAP nanoparticles loaded with drugs for local treatment. Due to the special structure on the surface of HAP, using this material to carry drugs can slow the release of drugs [47,52]. This method may not be applicable in antibacterial systems that require high concentrations for a short period. However, various substances, such as chitosan, can also be added to accelerate drug release [52]. Mondal et al. [53] also developed magnetic HAP to achieve controlled drug release. In this material, a core–shell structure was constructed with HAP as the shell and magnetic nanoparticles (Fe^2+^) as the functional core. The HAP shell leverages its excellent biocompatibility and osteointegration properties, while the magnetic core responds to external stimuli for targeted therapy and hyperthermia treatment. Once introduced into the bloodstream, the magnetic core enables the material to accumulate in regions with high magnetic field gradients under an external magnetic field. Additionally, the system responds to the acidic pH of the inflammatory microenvironment and releases drugs upon magnetic activation. The thermal effects generated by magnetic nanoparticles are also commonly utilized for antitumor therapy. However, HAP also involves certain drawbacks. The block-like HAP is hard and difficult to carve into a suitable shape, and the regenerated bone is difficult to grow into to form a stable connection; granular HAP lacks structural stability. Moreover, compared to PMMA, its mechanical strength cannot sufficiently ensure the stability of the affected area [54,55]. Some studies have incorporated metals, ceramic nanoparticles [54], graphene [55], etc. into natural HAP to improve its mechanical strength.

Currently, bioceramics are used as orthopedic scaffolds, as drug carriers for local drug delivery, and to prepare nanoparticles for local drug delivery. Bioceramics created from mesoporous silica are currently a hot research topic. Due to their mesoporous structure, these bioceramics are highly beneficial for cell growth and bone regeneration. Future research may focus on how to efficiently synthesize a large number of mesoporous bioceramic materials, enabling their wider application in the treatment of orthopedic diseases.

### 3.3 Polymers

**Fig. 2:**
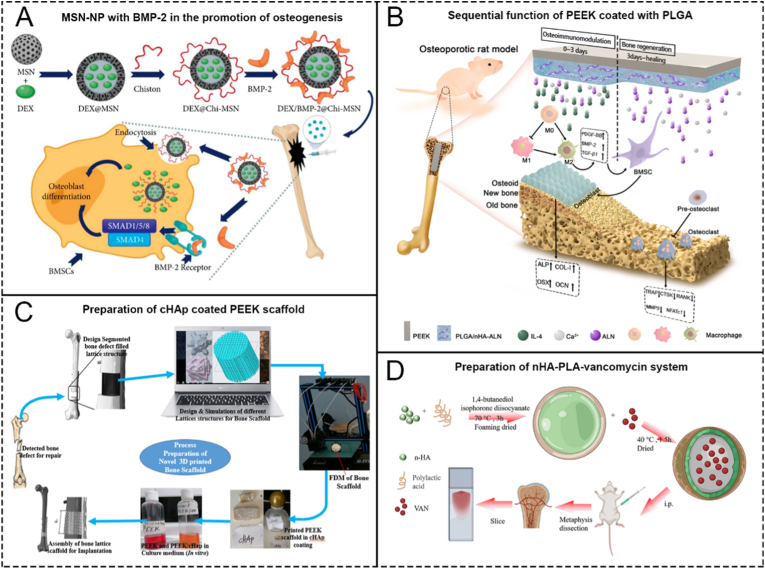
Schematics of 4 representative polymer biomaterials in the treatment of OM. A. Mesoporous Silica NP-Chitosan-Loaded BMP-2 in the Repair of bone defect in chronic OM. Reproduced with permission from Ref. [59]. B. Schematics showing PEEK coated with PLGA forming a programmed surface inducing osteoimmunomodulation and bone regeneration sequentially. Reproduced with permission from Ref. [61]. C. Schematics showing the preparation process of cHAp coated PEEK scaffold. Reproduced with permission from Ref. [57]. D. Schematics showing the preparation of nHA-PLA-Vancomycin system. Reproduced with permission from Ref. [66].

Traditional macromolecular polymers, such as PMMA bone cement, have been widely used in orthopedic implant materials. In recent years, with the flourishing development of polymer chemistry, some new types of polymer materials have emerged in the selection of materials for orthopedic implants, such as Polyetheretherketone (PEEK), Poly(lactic acid) (PLA), Poly(ε-caprolactone) (PCL), natural polymer materials such as Chitosan (CS) (Fig. 2A), Silk Fibroin (SF) [56], and PEEK composites like PEEK-hydroxyapatite (cHAp) [57], (Fig. 2C) etc. Polymers, when used as implants, have been demonstrated a series of advantages through practical applications, such as their chemical stability, slow, controlled drug release rates [58], enough biodegrability [59], and great mechanical ability, etc. The above characteristics determine why these materials are considered 'star' materials in the research of OM treating. The number of research papers on novel polymers loaded with various drugs for treating OM already demonstrates the popularity of these materials [60]. Below, we will focus on PEEK to introduce the application of these materials in the treatment of OM.

As an alternative to biomedical metals, PEEK is gaining more attention in orthopedics due of its outstanding attributes, such as excellent mechanical properties, inherent radiolucency, and sufficient biostability. More importantly, the elastic modulus of PEEK is similar to that of human cortical bone, thus circumventing the stress-shielding effects that destroy metallic implants and consequently avoiding peri-implant osteanabrosis [61,62]. The use of PEEK loaded with bioactive substances to treat OM is also being extensively studied. However, the biggest drawback of PEEK is its inherent biological inertness [63]. Due to this inertness, proteins are difficult to adsorb, and cell adhesion is also difficult; thus, integrating newly generated bone into the material as an implant is difficult. Surface coating or chemical modification of PEEK are two main methods used to overcome this problem. A detailed research is presented in Fig. 2B. Wang et al. [46] aimed to enhance the biocompatibility and osteogenic performance of PEEK by coating it with N-halogenated amine polymer. The material was constructed through a three-step process: First, micropores and oxygen-containing functional groups were created on the PEEK surface via concentrated sulfuric acid etching, followed by reduction with NaBH_4_ to provide grafting sites. In the second step, a polymer was grafted to introduce terminal amino groups. Finally, the material was reacted with sodium hypochlorite to form N-Cl groups, resulting in sp-PEEK-NCl. In aqueous environments, this material releases chloride ions, which exert a bactericidal effect on nearby bacteria. The N-Cl groups also provide contact-based antimicrobial activity. Over time, as chloride ions are consumed, the N-Cl groups revert to the original amine groups, restoring strong hydrophilicity and biocompatibility. This transformation promotes biomineralization and enhances local osteogenic capacity. (Fig. 1A). Wan et al. [64] studied the controllable sulfonation of PEEK using gaseous sulfur trioxide fumigation to reduce its inertness. The principle is to form a micro topological structure on its surface and increase the ability of proteins and other substances to adhere on the surface of PEEK. Similarly, Liu et al. [65] added a layer of zinc ions on top of the structure, further enhancing its ability to promote bone formation.

Given the obvious biologic inertness of PEEK, research on polymers with greater biocompatibility, such as Poly-(lactic acid) (PLA) and Poly-(lactic-co-glycolic acid) (PLGA), is also underway. Lv et al. [66] developed a nano-biomaterial based on nHA-PLA, loaded with vancomycin, for the local injection treatment of OM (Fig. 2D). This material not only exhibited antibacterial effects but also significantly promoted bone formation. Makiishi et al. [67]. utilized PLGA to load gatifloxacin (GLFX), and proved its significant bactericidal ability in OM model experiments.

### 3.4 Hydrogel

**Fig. 3:**
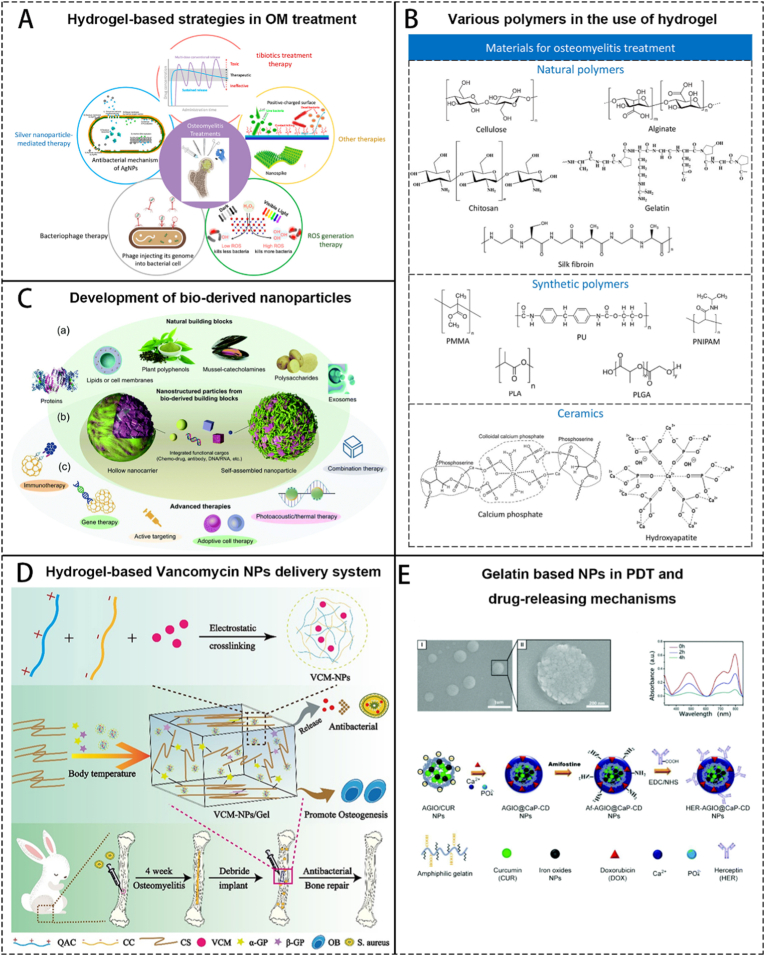
Schematics showing the basic information and utilizations of hydrogel and nanomaterials. A. Hydrogel based strategies in OM treating. Reproduced with permission from Ref. [30]. B. Various polymers in the use of hydrogel. Reproduced with permission from Ref. [30]. C. Schematics showing the development of bio-derived nanoparticle-related biomaterials in clinic use. Reproduced with permission from Ref. [70]. D. Hydrogel-based Vancomycin nanoparticle delivery system in OM treatment. Reproduced with permission from Ref. [68]. E. Gelatin based nanoparticles in the use of PDT and its drug-releasing mechanism. Reproduced with permission from Ref. [70].

As a special kind of polymer, hydrogels consist of large amounts of water and a network of cross-linked polymers. Common polymer materials included in hydrogel are presented in Fig. 3B. Due to their excellent ability of water content (typically 70–99 %), hydrogels are physically similar to human tissues; in addition, these materials exhibit good biocompatibility and can easily encapsulate hydrophilic drugs. Hydrogels are excellent delivery vehicles and have been used to treat a wide range of diseases, including tumor immunotherapy, wound healing, nerve regeneration, and bone repair (Fig. 3A). The physical and chemical properties of the hydrogel can also be modified by changing its cross-linking network structure, indicating that the material is highly plastic [30]. The shape of the OM affected part is irregular, and the hydrogel can obtain a highly consistent shape with the wound surface through its extremely high plasticity, which overcomes the shortcoming of some hard materials, which must be carved.

Hydrogel is not as strong as traditional polymers, so it cannot obtain as strong support capacity as PMMA, PEEK, bioceramics, etc. However, they are excellent drug delivery media due to their powerful drug-carrying capacity. These materials can also obtain inherent antibacterial properties through modification. Many studies have used hydrogels to wrap inorganic nanoparticles, antibiotics, etc. to achieve local delivery of drugs in the treatment of OM [68]. (Fig. 3D) However, the disadvantages of hydrogels cannot be ignored. Due to the high plasticity of hydrogels, the removal of residues often accompanies severe bone damage. The release rate of the drug itself is relatively unstable, making it difficult to maintain the local drug concentration. Hydrogels may also combine with local growth factors, resulting in blocked bone formation [69].

### 3.5 Nanomaterials

Nanomaterials differ from the implantable biomaterials commonly used in orthopedic surgeries because they have no elastic modulus and do not provide any physical supportive function. These materials are entirely used as 'Nanomedicine' or 'Nanocarriers'. They interact directly with the OM microenvironment by mixing with implants or through injection or application, producing a series of pharmacological effects to achieve precise treatment. The basic components of nanomaterials are nanoparticles, which serve either as the drug itself or as drug carriers. Below, we will provide a detailed introduction of nanoparticles.

Nanoparticles, with unique physical and chemical properties, have achieved a wide range of applications from diagnostics to drug delivery due to their diverse size, shape, surface chemistry, and potential for multiple functions (Fig. 3C). The multifunctional nanomaterial drug delivery system can load a large number of therapeutic drugs and simultaneously extend the circulation time of the drugs in vivo. Moreover, nanoparticles can be redesigned as environment-responsive drug delivery systems (such as pH, temperature, reactive oxygen species (ROS), magnetic fields, enzymes, radiation, ultrasound, etc.) to improve the therapeutic effects of drugs (Fig. 3E). In the treatment of OM, nanomaterials are often used as drug delivery tools and exhibit the following advantages compared to traditional delivery strategies: (1) increased drug solubility and stability; (2) drug release in a controlled or localized manner; and (3) biological barriers are overcome [70]. Besheli et al. [71] developed a scaffold-based system for localized and sustained release of vancomycin by encapsulating drug-loaded nanoparticles. The system consists of two levels of drug delivery carriers. At the first level, silk fibroin nanoparticles were formed via self-assembly through a desolvation method. These nanoparticles, bearing a negative surface charge, bind with positively charged vancomycin. At the second level, the drug-loaded nanoparticles are uniformly dispersed in a silk fibroin solution and fabricated into a macroporous scaffold using freeze-drying. This scaffold structure prolongs the drug release duration and results in a more gradual release profile. The system enables pH-responsive drug release at OM sites, as the silk fibroin scaffold matrix degrades under acidic conditions, leading to efficient drug release in the acidic inflammatory microenvironment. Similairly, Chen et al. [72] constructed a multifunctional nanocarrier carrying curcumin; and Wu et al. [73] designed titanium-based nanoparticles to act as fungicides under dark conditions. The nanoenzymes also belong to the category of nanomaterials, and their inherent enzyme catalytic activity provides new ideas for the treatment of OM, eliminating the shortcomings of natural enzyme substances, which are difficult to synthesize and require harsh storage conditions.

### 3.6 Scaffold coatings

**Fig. 4:**
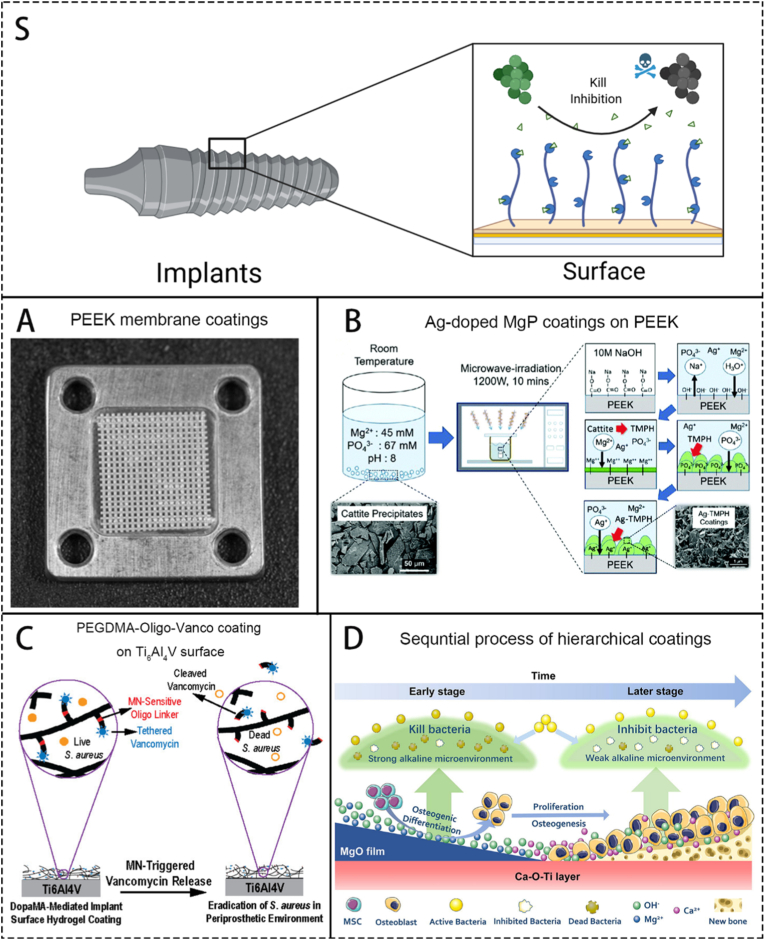
Photos and schematics of some representative scaffold coatings. S. A scheme of brief introduction of the working mechanisms of scaffold coatings. A. A stainless plate used to hold the PEEK membrane coatings. Reproduced with permission from Ref. [74]. B. Schematics showing the mechanism of the formation of Ag-doped MgP coatings on PEEK via microwave-irradiation. Reproduced with permission from Ref. [76]. C. Schematics of PEGDMA-Oligo-Vanco coating on Ti6Al4V surface and its antibacterial mechanisms. Reproduced with permission from Ref. [77]. D. Schematics showing the mechanisms of hierarchical coating producing antibacterial and osteogenesis process in a sequence. Reproduced with permission from Ref. [78].

Scaffold coatings do not refer to a specific type of biomaterial from a chemical structural perspective, but rather denote a class of biomaterials applied to orthopedic implant scaffolds to prevent implant-related infections. Currently, OM associated with orthopedic implants poses a significant threat to people's health. Pathogenic bacteria like SCV *S. aureus* can form biofilms on the surface of the scaffolds, and once formed, these biofilms are difficult for the body's immune system to completely eliminate [74], leading to pathogen colonization and the occurrence of OM. The reason why coatings are chosen instead of directly modifying the material to add new functions is that the modification process of some materials involves adding new substances or changing preparation conditions, which may cause the target material to lose its essential properties required for implantation (Fig. 4A) For example, during the preparation of PMMA mixed with antibiotics, the high temperature required for PMMA synthesis could destroy the biological activity of the antibiotics, resulting in poor antibacterial efficacy. By using coatings, the corresponding biological functions can be imparted to the surface of the implant material without affecting its inherent properties.

Initially, Scaffold Coatings primarily focused on preventing the formation of biofilms and preventing infections [75]. Nowadays, these coatings can also add other additional functions, such as incorporating growth factors to promote bone healing and adding new components to enhance biocompatibility (Fig. 4B) [76]. Diefenbeck et al. [75] applied a gentamicin coating to the outer layer of their self-developed macroporous titanium-oxide surface (bioactive TiOB). First, they generated a porous structure on the alloy surface through plasma chemical oxidation. The treated alloy was then immersed in a formic acid solution containing gentamicin and a fixative. Subsequent solvent evaporation allowed co-deposition of gentamicin and the fixative onto the alloy surface. This material enables rapid release of gentamicin during the early implantation phase, providing strong antibacterial efficacy. It also helps prevent bacterial adhesion and inhibit biofilm formation. In a rat OM model, the researchers compared gentamicin–sodium dodecyl sulfate (SDS) coatings and gentamicin–tannic acid coatings with negative and positive control groups, ultimately demonstrating that both gentamicin-based coatings yielded superior antibacterial effects compared to the control groups. Ghimire et al. [77]. developed a controllable drug-release coating triggered by micrococcal nuclease and loaded with vancomycin, known as PEGDMA-Oligo-Vanco (Fig. 4C). This coating links oligopeptides that are sensitive to S.aureus nuclease, enabling it to respond to infections caused by common pathogens in OM by precisely releasing vancomycin to achieve antibacterial effects. Trials in a mouse OM model using the coating on Ti_6_Al_4_V intramedullary nails demonstrated its reliable antibacterial capability and safe drug release behavior. Tan et al. [78] considered increasing biocompatibility and promoting new bone growth and cell adhesion through coatings. (Fig. 4D) The Ca-O-Ti and MgO layered coatings they developed can sequentially produce anti-infection treatment and osteogenic promotion effects. Studies in a rat OM model also confirmed this.

As biomaterials used for orthopedic implants, each material has its own elastic modulus to distinguish its hardness. Harder materials possess higher elastic moduli, making them less likely to deform noticeably under significant external forces, such as steel plates, titanium alloys, and some high-molecular polymers. When used as implants, they can better support and fix wounds, reducing secondary damage. Additionally, these materials can effectively transmit stress, promoting the healing of bone fractures and bone growth. Conversely, softer materials like bone cement and hydrogels have lower elastic moduli and deform easily under external forces, similar to the plasticity of modeling clay that children play with. This property means they cannot transmit stress nor provide any support. However, they can be used as wound dressings to exert their effects locally. For example, the good moldability of hydrogels allows them to closely conform to OM wounds or cavities, making the delivery of medication more thorough and comprehensive. Nanomaterials can be used as injectable medications or local dressings, interacting directly with OM wounds to achieve precise treatment.

In summary, the elastic modulus of biomaterials can demonstrate different treatment approaches and effects in the treatment of OM. Materials with different elastic moduli each have their own advantages and disadvantages, and accordingly, their uses vary. Our team believes that hard and soft materials can be used in stages during the treatment of OM, leveraging the therapeutic effects of materials with different elastic moduli at different stages of OM healing, to achieve comprehensive treatment across all stages and obtain superior therapeutic outcomes.

A brief conclusion of aforementioned biomaterials with antibacterial properties for OM is listed in Table 1 below.

**Table 1:** Biomaterials with antibacterial properties for OM.

Classification	Base materials	Cargos	Target bacteria	Other Biological function	Animal model	Ref.
Hydrogel	Chitosan	VCM	*S. aureus*	Anti-infection, promoting OB proliferation	Rabbit model	68
Hydrogel	Hematoma-like dynamic hydrogel	VCM and OGP	*S. aureus*	Anti-infection, promoting OB proliferation	Rabbit tibia model	79
Nanomaterial	Fibroin	VCM	MRSA	–	Male Wistar albino rats model	71
Scaffold coating	TiO_2_-SrHAP, Chitosan-Gelatin	VCM	MRSA	Anti biofilm-forming, promoting bone mineralization	–	80
Scaffold coating	PEGDMA-Oligo-Vanco	VCM	*S. aureus*	Anti biofilm-forming	Rodent femoral canal infection model	77
Bioceramic	HAp-VCM with Si-incorporated coatings	VCM,Si	*S. aureus*	Reduce side effects	Rat model	81
Polymer	PLGA 50:50 microbeads	Ciprofloxacin	–	Biodegradable	–	82
Polymer	PCL	Poloxamine, Ciprofloxacin	*Staphylococcus. spp*	Promoting OB proliferation	–	83
Polymer	PLA beads	Gentamycin	bacteria	Biodegradable	–	84
Polymer	p(SA:RA)s	Gentamycin	*S. aureus*	Biodegradable	Rat femoral OM model	85
Nanomaterial	Polydopamine functionalized MSN-core/shell	Ag nanoparticles, Dex	*S. aureus* *E. coli*	Enhancing osteogenic differentiation of cells	–	86
Bioceramic	Ca-incorporated NGp	Cu^2+^-NGp	*S. Aureus* *E. coli*	Promoting angiogenesis	Rat tibial OM model	87
Bioceramic	Multifunctional collagen-Bioactive glass	Cu^2+^	*S. aureus*	Promoting cell-mediated calcification and angiogenesis	Chicken embryo model	88
Nanomaterial	AuAg	Ag and Au NPs	*S. aureus*	Anti biofilm-forming	–	89
Nanomaterial	Au50	Au50	*S. aureus*	Induces M2 macrophage polarization, accelerates tissue repair	Rat femoral OM model	90
Nanomaterial	Au cage & monocyte	Aspirin	*S. aureus*	Programmed anti-inflammation	Mouse OM model	91
Bioceramic	BCP	Zn^2+^,ZnO	*S. aureus, E.coli*	Promoting osteogenesis	SD rats femoral OM model	92
Nanomaterial	Ag_2_O/TiON	TiO_2_'s PD effect,Ag^+^	*E. coli*	–	–	73
Polymer Bone cement	PMMA@polymer	HDP-mimicking peptide polymer	MRSA	–	Rabbit OM model	5

The characteristics of various biomaterials have shown unique advantages in the treatment of OM, such as sufficient mechanical strength, biocompatibility, and bone integration. Even if some materials do not possess certain advantages, they can be combined or modified with other materials to achieve the needed performance. The most important advantage of biomaterials is their powerful drug-loading ability. OM is caused by local bacterial infection and reproduction within the bone, producing toxins or other harmful substances that harm local bone tissue. Additionally, there is less angiogenesis and poor blood flow in the inflamed area, making it difficult to transport drugs through the bloodstream and achieve antibacterial concentrations in a local area. How to deliver sufficient medication to the local area of OM is the main research direction. By loading material with the necessary drugs and releasing drugs at the appropriate time, local treatment of OM can be achieved, overcoming adverse effects, such as high systemic side effects, and susceptibility to drug resistance. In addition, through some chemical or physical modifications, biological materials can also be endowed with one or more functions, such as electrical activity, microenvironment response activity, antioxidant/anti-inflammatory activity, immune regulatory activity, and osteogenic activity. Currently, the researches about using biomaterials to treat OM mainly focus on antibacterial treatment. Surely, it is of great importance to kill bacteria at the infection site, but taking the complicity of OM microenvironment into consideration, we conclude that antiinflammation, antioxidation, immunoregulation, promotion of angiogenesis and osteogenesis should also be taken into the researchers’ view. These aspects mentioned above is as important as antibacterial treatment in accelerating the healing of OM wounds.

Currently, there's no articles reviewing the contents in the classification method mentioned above. In our view, we think that by sorting articles in the way above, can make researchers, especially ones in the biomaterial field to have new perspectives and help them with their researches. So, in this review, we will briefly introduce the use of biomaterials with different biomedical activities in treating OM.

## 4 Antibacterial biomaterials

Since bacterial infection is the primary cause of OM, the majority of current biomaterials focus on how to eliminate local infections to explore methods for treating OM. Current mainstream research includes the use of inherent antibacterial biomaterials, or materials that carry antibiotics, metal ions, nanoparticles, and materials that generate antibacterial substances through external physical signal stimulation. Specific material introductions are provided below.

### 4.1 Inherent antibacterial biomaterials (IAB)

Some biomaterials themselves exhibit antibacterial properties, and their antibacterial mechanisms vary. Other than the biomaterials that act as a carrier of other drugs or bioactive molecules, inherent antibacterial biomaterials are also widely reported. At present, the IAB used to treat OM are mainly chitosan, cationic polypeptides are also used, and some special structures on the surface or in the body of plants or animals can protect wounds or perform antibacterial effects. These materials belong to the natural IAB, which means they can be found in the nature environment, without the need of undergoing any chemical modifications. Another kind of IAB is not naturally found, they underwent certain kinds of chemical modification to achieve inherent antibacterial activity, such as quaternization. Our cut-in point of IAB will be mainly through these 2 kinds of IABs.

#### 4.1.1 Natural IABs

Since every organism needs to develop resistance to pathogens during evolution, many organisms in nature possess their own antibacterial mechanisms. Most animals and plants generate unique biochemical substances to exert antibacterial effects, leading to research on the use of extracts from various plants for antibacterial treatments. Chitosan and cationic polypeptides obtained from nature are representative substances in this regard. We will focus on these two materials to introduce natural IABs.

Chitosan is a nontoxic, biological compatible, degradable natural cationic polymer known for its low immunogenicity, good antimicrobial and antioxidant effects, and significant wound-healing activity. Applying chitosan can reduce the formation of biofilms on the surface of implants and reduce secondary infections caused during the implantation process. We have confirmed in our previous studies that chitosan-based biomaterials possess good broad-spectrum antibacterial activity, many have demonstrated the effectiveness of chitosan in inhibiting the growth of various bacteria [[93], [94], [95]]. Li et al. [96] found that the antibacterial activity of chitosan results from amino protonation and cation formation of its molecular side chains in acidic solutions. Chitosan has also been demonstrated to have certain drug-controlled release capabilities, so most literature is studying the use of chitosan loaded with antibacterial active drugs for local treatment of OM. Radwan et al. [97] developed a chitosan-based calcium phosphate composite loaded with moxifloxacin hydrochloride for the local treatment of chronic OM. The material uses chitosan as the matrix, calcium phosphate as the inorganic filler and bioactive component, and moxifloxacin as the therapeutic agent against OM. Using an in-situ precipitation method, phosphate and calcium sources were deposited within the chitosan matrix. The resulting composite powder was then mixed with moxifloxacin and compressed into small implantable scaffolds. After implantation at the infection site, the material enables localized burst release of the drug, rapidly establishing a high local antibiotic concentration. The intrinsic antibacterial property of chitosan further disrupts bacterial cell membranes, producing a synergistic antimicrobial effect. Moreover, owing to chitosan's antioxidant characteristics, the cytotoxicity of moxifloxacin is significantly suppressed, thereby promoting tissue recovery and reducing side effects. The literature indicates that although chitosan itself has good osteogenic activity, biocompatibility, and degradability, its mechanical strength cannot meet the requirements of orthopedic implants. The team combined it with commonly used calcium phosphate bioceramics to increase its mechanical strength and filled it with moxifloxacin hydrochloride with clear antibacterial activity. In animal experiments, this material also achieved good therapeutic effects; thus, the material havs the potential to be promoted and used in clinical trials.

**Fig. 5:**
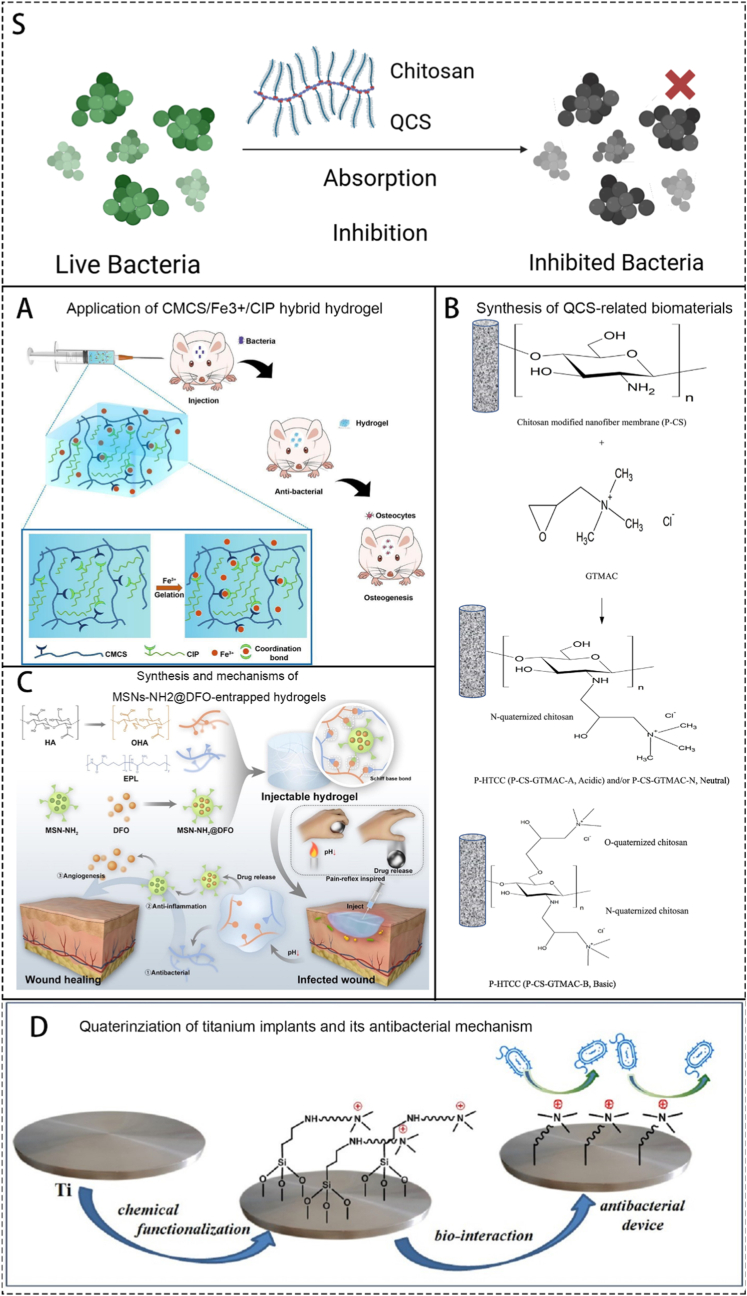
Schematics showing some utilizations of IABs in the treatment of OM. S. A scheme of brief introduction of current IABs and their mechanisms. A. Schematics showing the application of CMCS/Fe^3+^/CIP hybrid hydrogel in mouse OM model. Reproduced with permission from Ref. [105]. B. Synthesis of quaternized chitosan-related biomaterials. Reproduced with permission from Ref. [106]. C. Schematics showing the synthesis of MSNs-NH_2_@DFO-entrapped hydrogels and its healing mechanisms. Reproduced with permission from Ref. [103]. D. Schematics showing the quaterinziation of titanium implants and its antibacterial mechanism. Reproduced with permission from Ref. [107].

Cationic polypeptides are antimicrobial agents found naturally in living organisms where they participate in the innate immune response and defending mechanisms. Antimicrobial peptides (AMPs) are one of the many species of cationic polypeptides. They are usually short (∼10–50 amino acids), cationic (generally +2 to +9), and amphiphilic molecules that contain water-repellent residues [98]. In short, AMPs bind to negatively charged phospholipids on the outer layer of the bacterial wall through static electricity, and the water-repellent residues exposed during structural folding form pores in the bacterial lipid barrier, leading to overall instability and eventual dissolution of the cell membrane. This mechanism can be designed to combat antibiotic-resistant strains and can mobilize anti-inflammatory cells in the body through immune regulation, enabling the cells to play a more powerful role [99]. Previously, based on the antimicrobial principles of AMPs, we synthesized a series of N-arylimidazolium AMP mimics. These compounds have demonstrated effective antibacterial activity against multiple strains, along with low resistance rates, good blood compatibility, and low cytotoxicity [100]. Current researches focus on using other materials to carry newly synthesized peptides to the wounds (Fig. 5A). Zhao et al. [101] designed and synthesized a polylysine (EPL)/poly-ε-caprolactone (PCL) copolymer with good biocompatibility and biodegradability. Using EDC/NHS chemical conjugation, hydrophilic EPL was covalently linked to hydrophobic PCL to form an amphiphilic block copolymer. This copolymer self-assembles in an aqueous environment into nanoparticles with a core–shell structure, exhibiting a strong positive surface charge. The strongly positive surface enables electrostatic adsorption to bacterial cells, while also inducing oxidative stress and disrupting cellular metabolism, resulting in effective antibacterial activity. The copolymer readily disperses into nanoparticles and demonstrates stronger antibacterial effects than the copolymer alone. It exhibits broad-spectrum antibacterial activity against *E. coli*, *S. aureus*, and *Bacillus subtilis*. Yang et al. [102] designed a hydrogel that can continuously release antimicrobial peptides for up to 28 days and shows a strong antibacterial effect on *S. aureus*. Simultaneously, this hydrogel exhibits a good osteogenic effect and can promote bone regeneration. In our previous studies, we synthesized new bactericidal materials based on polypeptides. One of our studies [103] is inspired by the pain reflex, synthesized a kind of hydrogel that can reflect to local inflammation microenvironment, and achieved a sustained drug releasing pattern (Fig. 5C). At the same time, amino functionalized silica nanoparticles loaded with deferriamine (DFO) were captured on the hydrogel skeleton promotes local wound healing. Another study [104] prepared a tough conductive hydrogel using N-acrylylglycine amide (NAGA) and quaternium-salt chitosan-G-polyaniline (QCSP) as copolymers. It can be used to provide electrical stimulation around a wound. The polyaniline fragment can also play an inherent antibacterial role.

#### 4.1.2 Chemically modified IABs

As previously summarized, most biomaterials do not inherently possess antibacterial properties and require certain chemical modifications to gain intrinsic antibacterial effects. This might sound similar to the principle of using biomaterials to deliver drugs for the treatment of OM. However, the key difference lies in the fact that the molecules or substances used to modify these chemically altered biomaterials are directly bonded to the substrate via covalent bonds [107]. This creates a new substance through a chemical reaction during the production process. Typically, drug-loaded materials release drugs through the degradation or melting process of the carrier, allowing the drug molecules to interact with the inflammatory environment. In contrast, the antibacterial mechanism of these modified materials primarily involves the interaction of the modified molecules with the inflammatory microenvironment, without the degradation of the carrier, thus avoiding a series of side effects that could arise from the carrier degrading into the circulation, such as the risk of cardiovascular embolism caused by PMMA [108].

At present, the main method for chemically modifying IABs is quaternization (Fig. 5B). Among these, research on quaternized chitosan is in its maturity. Quaternized chitosan has been proven by many experiments to have antibacterial effects. Cheah et al. [106] fabricated an electrospun polyacrylonitrile (PAN) nanofiber membrane and modified it with quaternized chitosan. They first prepared PAN nanofibers via electrospinning, followed by alkaline hydrolysis to convert surface cyano groups into carboxyl groups, resulting in a P–COOH membrane. The membrane was then functionalized by immobilizing chitosan and subjecting it to quaternization under different pH conditions, yielding modified membranes with varying degrees of quaternization. The antibacterial mechanism of the material is attributed to electrostatic adsorption on its surface. The authors compared the antibacterial performance of these membranes with chitosan-coated nanofiber membranes having different quaternization levels, and demonstrated the strong antibacterial capability of quaternized chitosan based on its efficacy against *E. coli*. Wei et al. [109] developed a new anti-*S.aureus* hydrogel material based on quaternized chitosan and nanoparticles containing gentamicin, harnessing the antibacterial effects of both quaternized chitosan and gentamicin while avoiding the rapid drug release issue typically associated with drug-loaded hydrogels.

Besides quaternized chitosan, there are also studies on the chemical modification of other biomaterials, though the number of publications is relatively smaller. Celesti et al. [107] addressed the issue of orthopedic implant materials being prone to infection by designing a technique that modifies the surface of titanium alloy using quaternization (Fig. 5D). They used quaternary ammonium salts (QASs) and oleic acid (OA) to directly modify the surface of titanium alloy, giving it the ability to resist colonization by *E. coli*. Luss et al. [110] developed a polysaccharide gel and covalently bound antibiotics to glycan molecules, creating a gel with inherent antibacterial properties. This gel was used as a coating for some implants or porous materials and achieved good antibacterial effects in a rat OM model.

Unfortunately, there is hardly any literature on the use of chemically modified IABs in the treatment of OM, and currently, this area of research is largely unexplored. Our team believes that chemically modified IABs have demonstrated excellent antibacterial properties in multiple experiments. When natural IABs are difficult to obtain or insufficient in their antibacterial performance, chemically modified IABs can offer a new approach to treatment. Future research could focus on developing implant materials for OM treatment using chemically modified IABs, exploring their potential in this field.

### 4.2 Antibiotics

Antibiotics, as the main treatment for OM, have faced many problems. As mentioned in the introduction, the emergence of drug-resistant bacteria and undeniable side effects are increasingly limiting the application of antibiotics. With the goal of reducing the side effects caused by the systemic application of antibiotics, the use of biomaterials loaded with antibiotics for local treatment has become an emerging research direction. Biomaterials can achieve local controlled release, detect changes in the microenvironment caused by local inflammatory reactions, and thus achieve the goal of controlled drug release [111]. Below, we will discuss several commonly used antibiotics to illustrate the research on the combination of antibiotics and biomaterials in the treatment of OM.

#### 4.2.1 Glycopeptides

Glycopeptides are a class of antibiotics with a heptapeptide structure, demonstrating excellent bactericidal capability against anaerobic Gram-positive bacteria such as MRSA. This characteristic makes them commonly used in treating OM caused by MRSA infections [18]. However, their drawbacks are also evident; these antibiotics have relatively low concentrations in bones when administered systemically, meaning they have poor bone penetration. This necessitates very high systemic doses to achieve the minimum bactericidal concentration at the site of infection, leading to intolerable side effects. This has increasingly called for localized application of these antibiotics. There are numerous studies on the localized treatment of OM using biomaterials to deliver such antibiotics, mainly including vancomycin and teicoplanin. The number of cases of OM caused by MRSA is also significantly increasing [112]. Thus, glycopeptides are among the preferred drugs used to treat OM caused by MRSA. However, its strong side effects and the emergence of drug-resistant bacteria are increasingly limiting the application of glycopeptides. Moreover, due to the low bioavailability of glycopeptides, it is difficult to achieve effective concentrations in the affected area [113].

**Fig. 6:**
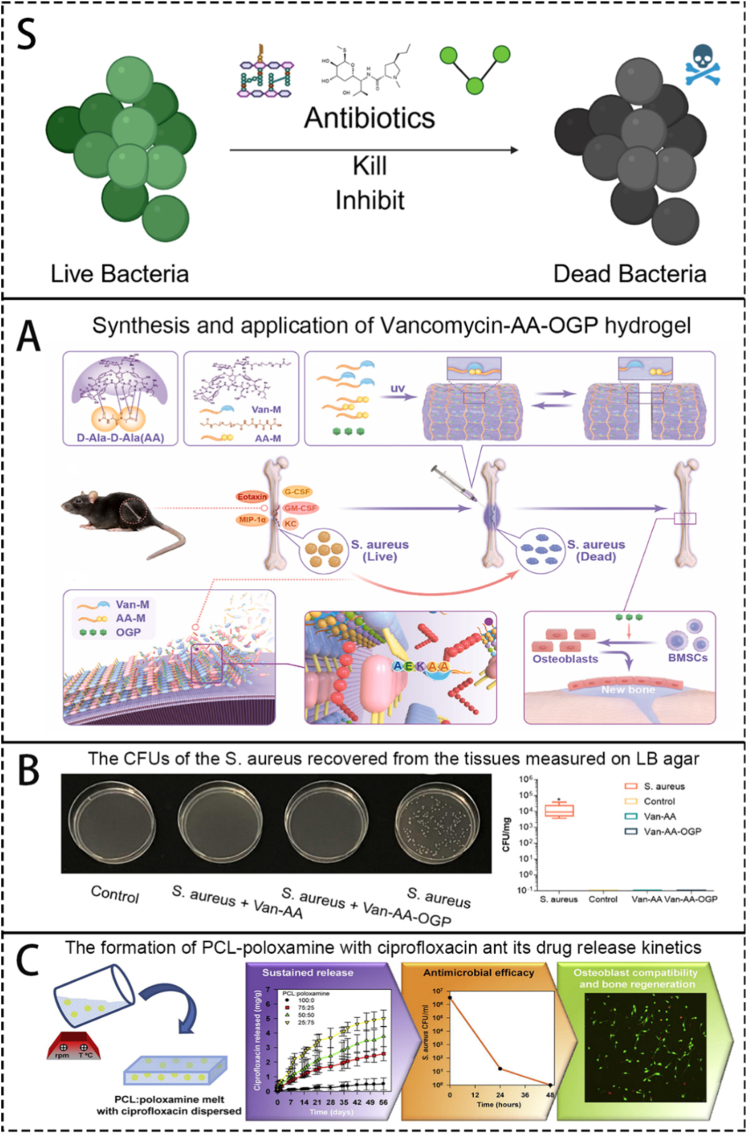
Schematics showing biomaterials in the delivering of antibiotics and evidences of their outstanding bactericidal effects. S. A scheme of brief introduction of currently used antibiotics and the mechanisms of healing OM. A. Schematics of the synthesis and application of Vancomycin-AA-OGP hydrogel and the mechanism of bactericidal and osteogenic effects. Reproduced with permission from Ref. [79]. B. The CFUs of the *S. aureus* recovered from the tissues measured on LB agar. Reproduced with permission from Ref. [79]. C. Schematics, charts and photos showing the formation of PCL-poloxamine with ciprofloxacin ant its drug release kinetics, bactericidal ability and osteoblast compatibility. Reproduced with permission from Ref. [83].

In the region of vancomycin, Tao et al. [68] prepared a chitosan-based thermosensitive hydrogel via electrostatic adsorption for generating vancomycin-loaded nanoparticles at local inflammatory sites. The nanoparticles are formed through electrostatic self-assembly between quaternized chitosan and carboxylated chitosan in an aqueous solution, effectively encapsulating vancomycin. These drug-loaded nanoparticles are then incorporated into the thermosensitive hydrogel, which serves as a delivery vehicle to the infection site. Upon temperature stimulation, the hydrogel releases both vancomycin and osteogenic components. In PBS, the drug release profile of this material meets the required therapeutic specifications. Wang et al. [79] combined vancomycin with a hydrogel to create a dynamic hydrogel system that simulates the hematoma environment (Fig. 6A and B). This system can reverse the inflammatory microenvironment caused by pathogenic bacteria such as *S. aureus*, providing a suitable physiological environment for bone healing. Ghimire et al. [77] noted that general loading methods may lead to excessive local antibiotic concentrations, leading to toxic effects on normal cells. They developed an oligonucleotide connector sensitive to *S. aureus* nuclease, which is loaded with vancomycin (PEGDMA-Oligo-Vanco). This material is used to create a coating and coated on implant materials such as Ti_6_Al_4_V. In the presence of *S*.*aureus*, vancomycin is released regularly and accurately to reduce local side effects. In our previous study [114], we synthesized vancomycin-conjugated silver nanoclusters (VSCs) to enhance the bactericidal efficiency of vancomycin against *S. aureus*. Further encapsulation of these nanoclusters within hydrogels improved their biocompatibility.

Teicoplanin-related researches in the treatment of OM are also undergoing. Chen et al. [115] synthesized a teicoplanin-loaded lipid liquid-crystalline (cubosomes) nanoparticle-laden gel to achieve sustained release of teicoplanin. This technology successfully reduced the concentration of teicoplanin in the blood and allowed for continuous local drug release for up to 36 days in OM wounds. Jia et al. [116] studied the application of using designated borate glass and chitosan (BGC) to deliver teicoplanin for the treatment of chronic OM. In vivo trials used a chronic OM model of New Zealand white rabbits infected with MRSA and observed good antibacterial and osteogenic effects. Lazarettos et al. [117] analyzed the efficacy of using biodegradable calcium phosphate salt carriers loaded with teicoplanin to treat OM caused by MRSA infections. They created an MRSA OM model in 36 New Zealand white rabbits, loaded the drug onto the carriers, and euthanized the rabbits at different time points to observe the recovery of OM at each time interval. The results demonstrated that this method of drug delivery showed promising efficacy for OM.

#### 4.2.2 Fluoroquinolones

Fluoroquinolones are the most used antibiotics in the oral therapy of OM due to its excellent bone penetrating ability [18], which means the antibiotic concentration in blood will not be too high while achieving minimum bactericidal concentration in OM tissue [83]. However, a considerably high dose of antibiotics and a long-time treatment should be given when treating OM orally, which may cause other issues including gastrointestinal side effects, non-compliant, etc. Research is ongoing regarding the use of biomaterials to deliver these antibiotics for the treatment of OM. Currently, widely used fluoroquinolones include ciprofloxacin, moxifloxacin, and levofloxacin. Among these, ciprofloxacin is the most commonly studied as a loaded compound. We will primarily focus on the research involving these three antibiotics to summarize the use of biomaterials delivering fluoroquinolones for the treatment of OM.

Ciprofloxacin, as a third-generation fluoroquinolone antibacterial drug synthesized, exhibits broad-spectrum antibacterial activity. It exhibits a bactericidal effect by affecting the reproduction of bacteria through key enzymes involved in DNA replication. And the cure rate for OM caused by Enterobacteriaceae pathogens is high [118]. Ramchandani et al. [82] developed a biodegradable PLGA-based biomaterial system for localized delivery of ciprofloxacin. The study prepared PLGA microspheres using two solvent systems: a non-polar method with dichloromethane–n-hexane, which yielded more porous and loosely structured gels, and a polar method using acetone–phosphate buffer, which produced denser microcapsule matrices. In the subsequent step, the drug-loaded PLGA microcapsules were consolidated via hydraulic compression to form macroscopic implants. The drug release mechanism of this system is distinctive, involving two primary phases: diffusion-dominated release and erosion-dominated release. The pH of the OM microenvironment modulates the solubility and ionization state of ciprofloxacin, thereby regulating the rate of the dissolution-based diffusion process. This study pioneered the study of biodegradable drug release materials and led subsequent research in this field. To achieve the goal of controlled release of ciprofloxacin, Puga et al. [83] considered modifying PCL materials that were economically affordable but exhibited a slow biological erosion rate and were not widely used in clinical practice (Fig. 6C). The team hoped to create a binary mixture material with appropriate viscoelasticity and drug release rate by mixing the substance with a poloxamine:PCL:poloxamine blend. In their study, PCL was used as the base carrier, and poloxamine was used as a bone regeneration promoter and accelerator of the release of ciprofloxacin. Better viscoelasticity, drug release mode, and erosion rate can be achieved by adjusting the ratio of the two.

Levofloxacin is also a third-generation like ciprofloxacin, which exhibits broad antibacterial spectrum towards aerobic bacteria. But its relatively strong toxicity restricts the extensive use of it. In contrast, moxifloxacin, the fourth-generation fluoroquinolone antibiotics have less toxicity and fewer side effects. Both of these antibiotics are researched in utilizing biomaterials combining with them when treating OM. Radwan et al. [97] investigated a chitosan–calcium phosphate composite for localized delivery of moxifloxacin hydrochloride (MOX) in the treatment of OM. This material not only releases MOX but also exhibits good biocompatibility, promoting the self-repair and regeneration of locally damaged bone tissue. The composite was prepared using an in-situ precipitation method: chitosan was first dissolved in acetic acid, followed by the addition of phosphate and calcium sources to form the nanocomposite. The resulting powder was then directly blended with moxifloxacin. Drug release from this material occurs primarily through a diffusion mechanism, enabling an initial burst release phase. Experiments conducted in a rabbit OM model further confirmed the favorable therapeutic efficacy of the material. Matos et al. [119] used calcium phosphate and bone cement as carriers for levofloxacin. They utilized Mg- and Sr-doped calcium phosphate (CaP) microspheres loaded into lactose-modified acrylic bone cement. This material has good mechanical strength and sufficient drug release capacity, maintaining antibacterial activity for up to 8 weeks. Fuglsang-Madsen et al. [120] introduced a novel technology named CarboCell, based on hydrophobic carbohydrate esters, triglycerides, and organic solvents. Upon entering an aqueous environment, the organic solvents leave the system, forming a depot that releases antibiotics over time. They used this depot to deliver levofloxacin and clindamycin for the treatment of implant-associated OM. A mouse OM model demonstrated that this material has the ability to kill *S. aureus*.

#### 4.2.3 Aminoglycosides

Aminoglycosides are mostly used to treat infections caused by gram-negative bacteria. The antibiotic is also used to treat OM and exhibits bactericidal ability against *E*. *coli*. However, due to its strong nephrotoxicity, high-dose systemic use may lead to irreversible consequences. The commonly used aminoglycoside antibiotics for treating OM are mainly gentamicin and amikacin. Streptomycin, as one of the earliest discovered aminoglycoside antibiotics, has fewer studies related to OM treatment, and those that exist are older and not timely. We will focus on gentamicin and amikacin to introduce the application of aminoglycoside antibiotics in the treatment of OM.

Gentamicin, as a star player among aminoglycoside antibiotics, is frequently studied. Loading gentamicin onto biomaterials can achieve local controlled release, reduce systemic drug concentration, and improve therapeutic efficacy. The use of gentamicin bead chain implantation for the treatment of chronic OM is among the preferred methods. The bead chain component is generally PMMA, which is not degradable and must be removed through secondary surgery after release. In addition, this method also involves a serious risk of secondary infection. Meyer et al. [84] developed a biodegradable bead chain based on poly-L-lactide (PLA) as a carrier for gentamicin, addressing the non-degradability limitation of PMMA bead chains. The material is fabricated using an innovative two-step process: first, gentamicin is transferred from the aqueous to the organic phase via hydrophobic ion pairing with a surfactant; then, both PLA and the drug are co-precipitated into microspheres through compressed antisolvent precipitation. Drug release occurs primarily through diffusion from the pores of the material. In addition to being biodegradable, this system enables significantly longer sustained drug release compared to PMMA bead chains, with an impressive cumulative release rate of up to 60 %—far exceeding the 10 % typically achieved by PMMA bead chains. Brin et al. [85] studied an injectable degradable polymer to release gentamicin. The team utilized polyanhydrides, which were extensively studied at the time, as the basis for biodegradable materials and explored the potential of injectable biodegradable poly(sebacic acid-castor oil-ester-anhydride), known as p(SA:RA), in delivering gentamicin for the treatment of chronic OM caused by *S. aureus*. By constructing a rat tibial OM model, the researchers verified the effectiveness of this polymer in treating OM.

Amikacin is also a popular choice among aminoglycoside antibiotics. It has great potential as an antibiotic for treating OM. However, there are currently fewer studies on the use of biomaterials to deliver amikacin for the treatment of OM. Luss et al. [110] developed a gel made from epichlorohydrin-activated hydroxyethyl starch (HES) and used it to covalently bind amikacin (HES-Am) to treat OM caused by implants. The preparation of this material involves a multi-step chemical synthesis process. First, HES is activated under alkaline conditions to introduce epoxy side chains while undergoing intermolecular crosslinking. Next, amikacin is covalently immobilized within the resulting gel to form a covalent complex. This complex is then infused into deproteinated and defatted bovine bone matrix via vacuum impregnation, and the final material is obtained after drying. The resulting product can be applied as a coating on implant surfaces or within the porous structure of trabecular bone, enabling comprehensive antibacterial coverage. Drug release is triggered by bacterial α-amylase, which specifically cleaves glycosidic bonds in the HES backbone, generating amikacin-carrying oligosaccharide fragments and thereby achieving targeted antibiotic release. Efficacy of the material was validated in a rat OM model. Similarly, Harrison et al. [56] built upon the basis of chitosan carrying antibiotics by adding mannitol to the base material to increase bacterial sensitivity to antibiotics. They also used this material to carry amikacin to test the antibacterial effect and compared it with other groups.

Supplementary information: Lipopeptides.

Lipopeptide antibiotics, characterized by their special structure similar to lipids and peptide chains, possess both lipophilic and hydrophilic properties. Daptomycin, as a representative antibiotic of lipopeptides, is widely used in the systemic treatment of OM. There are numerous studies on the systemic use of daptomycin for treating OM, but very few researchers have explored the use of biomaterials to deliver daptomycin for local treatment. We believe this is a promising research direction and hope that our summary can provide researchers with some new ideas.

#### 4.2.4 Drug release kinetics’ improvements through biomaterials science

Above concludes our summary of commonly used antibiotics delivered via biomaterials for the treatment of OM. These antibiotics were once the preferred choice for systemic treatment of OM. Due to the increased bacterial resistance caused by the overuse of antibiotics, traditional methods are becoming inadequate. Therefore, the idea of using biomaterials to deliver antibiotics for localized and precise treatment emerged. To achieve this target, Research of certain antibiotic-release kinetics of biomaterials has become a must. Sustained and on-demand release kinetics of antibiotics are 2 of the most widely accepted release kinetics, which significantly reduced the concentration of drugs in circulation, decreased side effects, and simultaneously increased the local concentration of antibiotics.

**Fig. 7:**
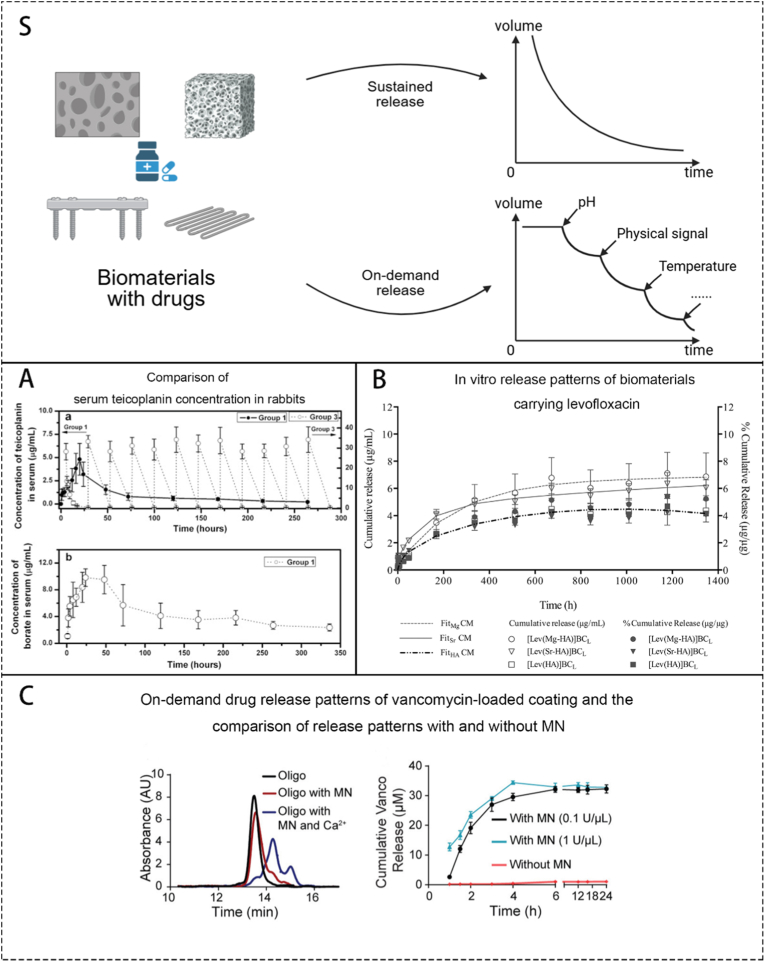
Different drug release patterns of currently researched antibiotic-carrying biomaterials. S. A scheme of brief introduction of mainly used drug release patterns. A. The comparison of serum teicoplanin concentration in rabbits treated by implantation TBGC pellets and by intravenous injection (upper) and serum concentration of borate (lower). Reproduced with permission from Ref. [116]. B. In vitro release patterns of biomaterials carrying levofloxacin. Reproduced with permission from Ref. [119]. C. On-demand drug release patterns of vancomycin-loaded coating and the comparison of release patterns with and without MN. Reproduced with permission from Ref. [77].

Sustained-release kinetics can lower the drug-releasing rate, which means drug will not be exhausted in a very short period of time, thus maintain a certain level of local antibiotic concentration for a longer period (Fig. 7A,B). Chen et al. [115] successfully achieved sustained drug release in their teicoplanin-loaded cubosomal gels, resulting in lower serum drug concentrations compared to intravenous administration. Tao et al. [68] also achieved sustained drug release over 25 days in their study by using nanoparticles or gels loaded with VCM. In our previous researches [121,122], we also attempted to use surface modification techniques to encapsulate antibiotics within hydrogel coatings or superhydrophilic nanocoatings on medical devices. This approach aimed to reduce the risk of hospital-acquired infections caused by bacteria on the surfaces of medical devices. Experimental results showed that the surface coatings modified using biotechnological methods could exhibit safe and effective antibacterial properties in a low-dose-dependent manner.

On-demand release biomaterials can sense the existence of local bacteria by interacting with their metabolites or markers, starting a series of reactions to release drugs, which means it can release drugs the same time pathogen invades, reducing side effects (Fig. 7C). Ghimire et al. [77] developed a vancomycin-loaded coating for intramedullary nails that can respond to Micrococcal Nuclease produced by *S. aureus*, achieving local and controlled release of vancomycin. Its antibacterial effect was confirmed in a mouse femur OM model.

In summary, research on using biomaterials to deliver antibiotics for the treatment of OM represents a significant advancement in the field of OM treatment.

### 4.3 Metals

Metals are also widely used as antibacterial agents in the treatment of various infections [123,124]. The treatment of OM mainly relies on ion/nanoparticle release, contact reactions, and catalytic mechanisms. Some metals play an important role as components of physically antibacterial sensitive materials. Due to the direct nature of this principle, it is not easy for bacteria to develop resistance. However, excessive exposure to metal ions can also cause damage to normal cells. Therefore, the controlled release of metal ions is also very important. The research on biomaterials for the treatment of OM with metal is a highly promising direction.

In current research, the use of biomaterials to deliver metallic substances for the treatment of OM primarily involves metal nanoparticles and metal ions. Delving into their common mechanisms, they all work by locally releasing metal atoms or ions, generating or catalyzing various biochemical reactions, thereby interfering with the normal physiological processes of pathogens to produce antibacterial effects [125]. For example, Due to the presence of metalloenzymes in cells, cells themselves have the ability to transport metal particles with similar properties. When metal ions used as drugs are mistakenly recognized and absorbed by cells, they cannot be metabolized by the cells, thereby interfering with normal cellular activities and producing cytotoxicity. For example, most metal nanoparticles have a large catalytic surface area, which can generate a large amount of reactive oxygen species (ROS), damaging the biomolecules essential for cell survival. Metal nanoparticles can also interfere with cell survival by disrupting cellular metal transport pathways.

Common metallic materials used for treating OM include intrinsic antibacterial metals that can produce antibacterial effects without external stimuli, and some metallic materials that require physical signals from the external environment to generate antibacterial effects. Below, we will introduce these materials from these two aspects.

#### 4.3.1 Metals with inherent antimicrobial effects

Literature involved in this part are the use of certain metals without the need of external stimuli. They can produce antibacterial effects as soon as they are dispersed into the OM microenvironment. We will introduce this part through the most widely used metals in antibacterial applications. The metals we include mainly include noble metals, copper, zinc and titanium.

**Fig. 8:**
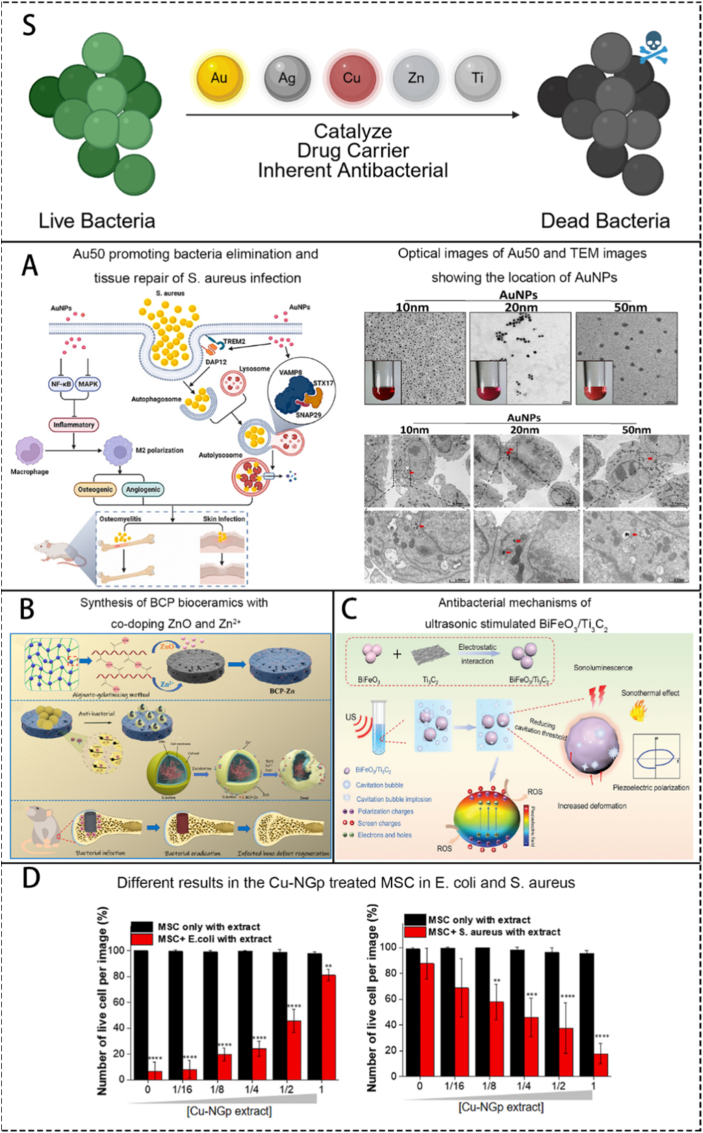
Schematics and charts showing the mechanisms of various metal biomaterials in OM treatment and their notable healing effects. S. A scheme of brief introduction of currently used metals in the use of treating OM. A. (Left) Schematic illustration of Au50 promoting bacteria elimination and tissue repair of *S. aureus* infection. (Right) Optical images of Au50 and TEM images showing the location of AuNPs inside the cells. Reproduced with permission from Ref. [90]. B. Schematics of the synthesis of BCP bioceramics with co-doping ZnO and Zn^2+^ and its antibacterial and regenerating mechanisms. Reproduced with permission from Ref. [92]. C. Schematics of antibacterial mechanisms of ultrasonic stimulated BiFeO_3_/Ti_3_C_2_. Reproduced with permission from Ref. [133]. D. Different results in the Cu-NGp treated MSC in *E. coli* and *S. aureus*. Reproduced with permission from Ref. [87].

Silver, as a long-standing antibacterial agent, exhibits obvious antibacterial effects. Silver ions (Ag^+^) released by active agents react with peptidoglycan in the cell wall, resulting in the destruction of the cellular skeleton such as bacterial cell walls and exerting an antibacterial effect. Gold atoms are commonly used as antibacterial agents in their nanoparticle form. Gold nanoparticles (AuNPs) have also drawn much attention due to their biological inertness and easy surface functionalization (Fig. 8A). Lee et al. [89] investigated the favorable properties of noble metal nanoparticles in combating excessive inflammatory responses and developed Au–Ag nanocomposites, demonstrating their low toxicity, good biocompatibility, and local anti-inflammatory effects. The material was synthesized using chloroauric acid, silver nitrate, polyethylenimine, and tannic acid as precursors. The formation process involved three main steps: mixing, co-reduction and nucleation, followed by self-assembly and stabilization, resulting in a unique pearl-necklace-like network structure. Tannic acid coats the surface of the material, forming a protective crown. This composite exhibits remarkable antioxidant properties, where the intrinsic antioxidative capacity of gold atoms synergizes with the antioxidant activity of tannic acid, enabling effective scavenging of local ROS. This process interrupts the propagation of inflammatory signals and significantly downregulates the expression of inflammation-related signaling molecules, thereby suppressing inflammation at its source. In our previous studies, we also synthesized silver nanoclusters (AgNC) successfully and it showed broad-spectrum antibacterial activity. Our team [114] grafted 3-carboxyphenylboric acid onto the molecular skeleton of gelatin and crosslinking it with polyvinyl alcohol, and coated vancomycin-silver nanoclusters(VAN-AgNC) with Nimesullide(NIM) onto it, which showed great antibacterial activity. In our previous studies [94,126,127], silver ions and nanoparticles both showed significant antibacterial activity.

Copper, whether in the form of metals or compounds, exhibits broad-spectrum antibacterial, anti-inflammatory, and anti-proliferative properties and has been widely used as an antibacterial element in orthopedics [[128], [129], [130]]. It also participates in the metabolism of normal cells and plays a unique catalytic role in the biological system, promoting the formation of collagen in bones, blood vessels, and skin. Some researches incorporated copper ions into orthopedic implants to prevent OM and promote regeneration [130]. (Fig. 8D) Seo et al. [87] aimed to achieve targeted delivery of copper ions to inflammatory sites while minimizing potential side effects. They developed copper-doped calcium silicate-based bioactive nanoglass (Cu-NG) particles and investigated their osteogenic, angiogenic, and antibacterial properties. The material was prepared by incorporating copper into silicate-based nanoglass particles, forming Cu-NG. The resulting powder was then mixed with a monosodium phosphate solution to produce an injectable, self-setting paste. At the inflammatory site, the incorporated copper ions are released in a sustained manner, inducing ROS generation within bacterial cells and exerting antibacterial effects. Additionally, silicate and copper ions act synergistically to upregulate VEGF expression, thereby promoting angiogenesis. The calcium ions inherently present in the glass further support bone formation. These multifunctional effects were validated in a rat OM model. Ryan et al. [88] also noted the excellent wound healing and osteogenic properties of copper ions. They mixed bioactive nanoglass with multifunctional collagen and copper ions to create a scaffold structure with good mechanical properties and attained good antibacterial activity against *S. aureus*. Its good biocompatibility and osteogenic activity were also confirmed through chicken embryo experiments.

Zinc plays an indispensable role in bone metabolism by stimulating osteoblasts and inhibiting osteoclasts [131]. Due to the biocompatibility and osteogenic activity of zinc, it is a feasible new choice for orthopedic implant materials (Fig. 8B). More importantly, Zn^2+^ has been demonstrated to have significant antibacterial and osteogenic properties [132]. He et al. [92] incorporated zinc ions and zinc oxide into biphasic calcium phosphate (BCP) ceramics to directly impart antibacterial and osteogenic properties. The material was synthesized using an alginate gel-based method: BCP powder was uniformly dispersed in a sodium alginate and hydrogen peroxide solution to form a slurry, which was then poured into a zinc chloride solution to produce a crosslinked hydrogel. The resulting gel was finally converted into ZnO- and Zn^2+^-co-doped BCP ceramics through high-temperature sintering. The material exerts antibacterial effects via multiple synergistic mechanisms, including Zn^2+^-induced disruption of bacterial cell membranes and interference with key enzymes, as well as ZnO-triggered generation of ROS. Additionally, Zn^2+^ stimulates bone marrow mesenchymal stem cells, promoting their differentiation into osteoblasts and thereby accelerating bone regeneration.

Titanium alloy has been widely used in the manufacturing of stent materials for internal implantation due to its excellent biocompatibility and mechanical properties. Due to the lack of strong antibacterial activity of titanium alone, most studies have focused on using titanium alloys to carry other antibacterial substances into the site of OM for treatment or to prevent implant-related infections. Wu et al. [73] prepared nitrogen–silver co-doped titanium dioxide nanoparticles (Ag_2_O/TiON), which exhibit significant antibacterial activity against *E. coli* under visible light irradiation at wavelengths longer than 400 nm. The material was synthesized via a sol–gel method, in which a titanium source and a nitrogen source were dissolved together in ethanol, followed by the introduction of a silver precursor. The mixture was then gelled, dried, and calcined to obtain the final nanopowder. Nitrogen doping narrows the bandgap of TiO_2_, enabling the material to absorb visible light. Meanwhile, Ag_2_O acts as an electron trap, prolonging the lifetime of photogenerated holes and thereby enhancing photocatalytic activity. This synergistic effect promotes the generation of more free radicals, leading to more severe damage to bacterial cells.

#### 4.3.2 Metals that need physical stimulation to produce antimicrobial effects

Some metals itself do not produce any antibacterial effects since they do not interfere with cells’ life cycles. But when given foreign stimulation like ultrasonic wave, microwave or photon, they can be stimulized and release antibacterial substances, mainly ROS (Fig. 8C). Li et al. [133] developed a ferroelectric ultrasonic interface which can be triggered by ultrasound and then exhibit ROS generating ability, Zhu et al. [134] also mentioned the ROS generating under microwave stimulation in their molybdenum and tungsten oxides. Detailed description and literature of this part is contained in section 4.4.

### 4.4 Antibacterial molecules with other specific functions

Against the backdrop of poor antibiotic application, strong side effects, and bacterial resistance, other functionalized antibacterial molecules were explored and found that physical methods for the antibacterial treatment of OM have also become a promising research direction. These molecules can respond to external signals and perform other specific biochemical functions like catalyzing or drug-releasing. Currently, the most widely used Physical therapy includes ultrasound, microwave, photothermal and photodynamic therapy. They act by interfering with sensitizers implanted into the OM wounds. Sensitizers receive signals and generate bactericidal substances such as ROS and free radicals, and achieve bactericidal effects. There are also researches focusing on the heat generating method, utilizing microwave to produce bactericidal-heat. Biomaterials in the region mentioned above have a relatively large amount of research and review, so we will briefly introduce some of the representative researches from 3 aspects: photon-triggered, sound-triggered and microwave-triggered biomaterials. Additionally, multi-stimulus responsive biomaterials also play an important role in current researches. These materials respond to several kinds of external stimulus. This kind of biomaterials shows a promising developing talent in tailored treatment of diseases.

#### 4.4.1 Photon-triggered biomaterials

**Fig. 9:**
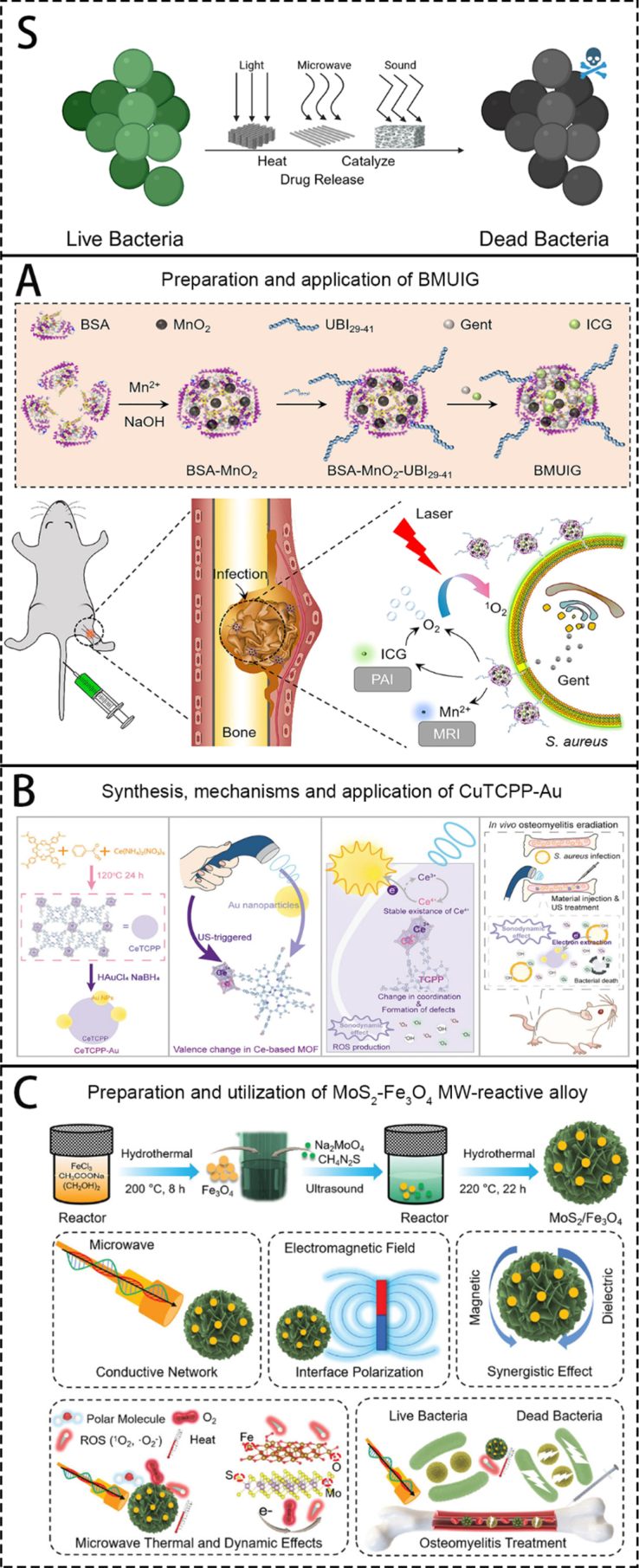
Schematics of representative biomaterials with specific functions. S. A scheme of brief introduction of currently used biomaterials utilizing physical signals. A. The preparation of BMUIG and its application in photodynamic therapy of OM. Reproduced with permission from Ref. [136]. B. Schematics of the synthesis of CuTCPP-Au sonosensitizer, its antibacterial mechanisms and its application in treating OM on mouse model. Reproduced with permission from Ref. [140]. C. Preparation steps of MoS_2_-Fe_3_O_4_ MW-reactive alloy and schematic illustration of its excellent MW absorption ability for treating OM. Reproduced with permission from Ref. [145].

Photodynamic therapy and photothermal therapy were initially used to treat cancer, which are also the two main functions of photon-triggered biomaterials. Recently, research on these two therapies for the treatment of bacterial infectious diseases has also been in full swing. Photodynamic therapy involves using photosensitive materials or drugs to produce catalytic reactions or bactericidal substances such as reactive oxygen species after local exposure to light waves [135]. Photothermal materials, on the other hand, mainly release heat, increase the temperature of the surrounding environment, increase the sensitivity of bacterial cells to drugs, and increase the release of drugs, thereby achieving better bactericidal effects on bacteria (Fig. 9A). Lu et al. [136] synthesized a bacteria- and inflammation-targeting multifunctional nanodrug, designated as BSA–MnO_2_–ubiquitin_(29_–_41)_–indocyanine green (ICG)–gentamicin (BMUIG), for combined antibiotic and photodynamic therapy of OM. The material was constructed by biomineralizing MnO_2_ onto bovine serum albumin (BSA) under alkaline conditions to form nanoparticles, followed by chemical conjugation of ubiquitin_(29_–_41)_ peptide. Finally, both ICG and gentamicin were loaded onto the nanoparticles via physical adsorption, yielding the BMUIG complex. This drug system serves as an adjuvant for photodynamic therapy, in which manganese dioxide catalyzes the conversion of hydrogen peroxide to ROS at an accelerated rate, resulting in enhanced bactericidal efficacy. Our previous studies have also successfully constructed photothermal bactericidal systems. Guo et al. [137] developed a superoxide dismutase (SOD) simulation center with photothermal anti-infection ability, and created a wound healing hydrogel. The results of in vitro experiments showed that the hydrogel had the ability of anti-infection by scavenging reactive oxygen species. These abilities promoted the transformation of macrophages into M2 and restored the inhibitory effect of infection on the proliferation and migration of fibroblasts.

#### 4.4.2 Sound-triggered biomaterials

Sound-triggered biomaterials mainly applies the inherent physical effects of ultrasound, as well as various bactericidal substances produced when combined with sound-sensitive materials, to generate antibacterial effects [138]. Among them, sound-sensitive materials may generate catalytic activity and produce substances, such as reactive oxygen species, that cause bactericidal effects when receiving sound stimulation. At present, precious metals, piezoelectric materials, porphyrins, and their derivatives have been used as sound-sensitive agents (Fig. 9B). Li et al. [133] developed a BiFeO_3_/MXene (Ti_3_C_2_) composite that utilizes ferroelectric–ultrasonic interfacial engineering activated by ultrasound. The material was constructed via electrostatic self-assembly, where the positively charged surface of BiFeO_3_ nanoparticles and the negatively charged surface of MXene nanosheets attract each other, resulting in a BiFeO_3_-encapsulated MXene structure with a formed Schottky junction. Under ultrasonic mechanical stimulation, the material exhibits piezoelectric and cavitation effects, generating electric fields and electron–hole pairs. The MXene component efficiently transfers photogenerated electrons, which reduce surrounding oxygen to produce superoxide anions that damage bacterial cellular components. This system demonstrates strong catalytic activity and an exceptionally high reactive oxygen species (ROS) yield under ultrasound irradiation. Zeng et al. [139] also based on enhancing the catalytic activity and efficiency of sound sensitizers. Zheng et al. [140] prepared cerium-based MOFCeTCPP using tetra (4-carboxylphenyl) porphyrin (TCPP) and (NH4)_2_[Ce(NO_3_)_6_] as solvothermal reactions. TCPP was used as a sound sensitizer, and a cerium-based MOF was modified with gold nanoparticles to improve the catalytic activity of the MOF. Under ultrasound irradiation, gold nanoparticles can convert 1/3 of Ce^3+^ into Ce^4+^, changing the coordination number of Ce and hindering electron-hole recombination, thereby maintaining stronger catalytic activity and generating more reactive oxygen species. In vitro and in vivo experiments have also demonstrated the high catalytic activity of this substance. Cheng et al. [141] synthesized ultrasmall platinum copper alloy nanoparticles (PtCuNPs) modified with polymaleic anhydride octadecene polyethylene glycol (C_18_PMH-PEG), which possess strong catalytic activity, high sound sensitivity, and glutathione consumption ability. Under ultrasound, this substance can catalyze the generation of reactive oxygen species, and it can also consume a large amount of local glutathione, clearing obstacles for the sterilization of reactive oxygen species. The novel point is that this material also exhibits catalytic effects similar to Fenton reactions.

#### 4.4.3 Microwave-triggered biomaterials

Microwaves exhibit several advantages, including strong penetration, high heating efficiency, and few side effects. Due to its ability to centrally heat a certain location, its thermal antibacterial effect has strong targeting and broad prospects in the treatment of deep tissue infections [142]. Microwaves can stimulate the catalytic activity of microwave-sensitive materials and generate reactive oxygen species through irradiation (Fig. 9C). At the same time, the thermal effect of microwaves can increase local temperature and increase the sensitivity of bacterial cells to drugs. Zhu et al. [134] investigated microwave-sensitive MoO_2_–WO_3_ alloys. MoO_2_ exhibits metallic conductivity, providing free electrons and electrical conduction, while WO_3_ offers abundant oxygen vacancies that act as charge traps to enhance carrier concentration. The researchers synthesized MoO_2_–WO_3_ heterojunctions via chemical vapor deposition, forming an intimately connected structure, and treated the material with sodium borohydride to introduce a high density of surface oxygen vacancies. Owing to the conjugated polarization effect and the presence of oxygen vacancies in this configuration, the material simultaneously exhibits strong microwave catalytic and microwave thermal properties. Under microwave irradiation, the material undergoes dielectric polarization, converting microwave energy into thermal energy. The heterojunction structure enables microwave responsiveness, enhancing oxygen adsorption and catalytic capacity to generate singlet oxygen. This catalytic effect synergizes with the microwave thermal effect to achieve efficient antibacterial activity. Similarly, multiple networks, such as molybdenum disulfide and carbon nanotubes, developed by Jin et al. [143] enhances these two effects through multiple reflections and scattering. Wei et al. [144] placed the center around the Fenton reaction. They used Prussian blue doped with sodium ions as a microwave-responsive material, releasing iron and ferrous ions while generating thermal effects. The thermal effect promotes the release of ions and can also increase the permeability of bacterial cell membranes, thereby increasing the number of ions entering the cell and achieving a killing effect. Incorporating sodium ions into this material can make the release of iron and ferrous ions irreversible, further increasing the release ability.

A brief comparison table of the aforementioned materials is presented in Table 2 below.

**Table 2:** Comparisons between various kinds of antibacterial biomaterials.

Antibacterial agents	Antibacterial mechanisms	Anti-biofilm forming	Intracellular Bactericidal	Carrier/Form	Sensitive strain	Ref.
CS-Based Calcium phosphate scaffolds loaded with MOX	Moxifloxacin local sustained release	Not mentioned	Not mentioned	Polymer (CS)	*S. aureus*	97
Cationic EPL-conjugated polymeric nanoparticles	EPL absorption damage to membrane	Not mentioned	Not mentioned	Polymer (PCL) Nanomaterial (NPs)	*E. coli*, *S. aureus,* *B. subtilis*	101
Quaternized CS modified nanofiber membrane	Quaternized CS bind with negatively charged membrane	Not mentioned	Not mentioned	Polymer (PAN)	*E. coli*	106
Functionalized Ti disc with QASs	QASs bind with negatively charged membrane	Yes	Not mentioned	Scaffolds (Ti)	*E. coli*, *S. aureus*	107
Teicoplanin-loaded lipid liquid-crystalline NPs laden gel	Teicoplalnin local sustained release	Not mentioned	Not mentioned	Nanomaterials (Crystalline NPs)	*S*. *aureus*	115
PLGA 50:50 implants loaded with ciprofloxacin	Ciproflo.xacin local sustained release	Not mentioned	Not mentioned	Polymers (PLGA)	*S. aureus, S. epidermidis, P. aeroginosa*	82
Levofloxacin-loaded acrylic bone cements (PMMA)	Levofloxacin local sustained release	Not mentioned	Not mentioned	Bone cement (PMMA)	*S. aureus, S. epidermis, E. coli*	119
MN-Triggered On-Demand Release of VAN from IM Implant	Vancomycin local controlled release	Yes	Not mentioned	Scaffold coatings	*S. aureus*	77
TNA-capped AuAg nanocomposites	Ag & Au NPs produce ROS	Yes	Yes	Nanomaterials (NPs)	*S. aureus*	89
Nanoglass paste made of ∼200-nm-sized silicate-glass (with Ca, Cu) particles--Cu-NGp	Ca and Cu ions local release, elevating ROS level locally.	Not mentioned	Not mentioned	Nanomaterials (Nanoglass particles)	*E. coli*, *S. aureus*	87
ZnO and Zn^2+^ doped BCP bioceramics	Zn generates ROS, inactivates proteins and deposits	Yes	Not mentioned	Bioceramics (BCP scaffolds)	*E. coli*, *S. aureus*	92
Titanium dioxide NPs codoped with Ag_2_O/TiON	Ag_2_O/TiON NPs, under visible light irradiation, produce ROS	Yes	Not mentioned	Nanomaterials (NPs)	*E. coli*	73
Bovine serum albumin-MnO_2_-ubiquicidin_29-41_-indocyanine green-GEN	Photon-triggered local production of ROS and release of GEN	Not mentioned	Not mentioned	Nanomaterials	*S. aureus*	136
BiFeO_3_/MXene (Ti_3_C_2_) nanoparticles triggered by ultrasound	Ferroelectric polarization enhances the catalytic act, produces more ROS	Yes	Not mentioned	Nanomaterials (NPs)	*S. aureus*	133
CeTCPP decorated with AuNPs	CeTCPP helps AuNPs under ultrasound irritation produce ROS	Yes	Not mentioned	Nanomaterials (NPs)	*E. coli*, *S. aureus*	140
PtCu NPs nanosensitizer modified with C_18_PMH-PEG	US induced fenton-like reaction generate ROS, deplete GS.	Yes	Yes	Nanomaterials (NPs)	*E. coli*, *S. aureus*	141
MW-responsive composite heterojunction of MoO_2_/WO_3_	MoO_2_ and WO_3_ oxygen vacancies enhance catalytic act produce ROS	Not mentioned	Not mentioned	Nanomaterials	*S. aureus*	134
Na^+^ inserted metal-organic framework	MW strengthened Fenton reaction and thermal effects	Not mentioned	Yes	Nanomaterials (nanocubes)	*E. coli*, *S. aureus*	144
GEN coating of plasma chemical oxidized titanium alloy	GEN elutes from the surface, producing antibacterial effect.	Yes	Not mentioned	Scaffold coatings	*S. aureus*	75
Cationic Starch Modified Curcumin	CS@Cur target bacteria cell wall and produce ROS under US	Not mentioned	Yes	Nanomaterials (NPs)	*E. coli*, *S. aureus*	72

#### 4.4.4 Multi-stimulus responsive materials

As a research hotspot in materials science in recent years, "multi-stimuli-responsive materials" have gained significant favor among materials scientists [146]. These materials can produce corresponding responses (e.g., shape changes, permeability alterations, etc.) to various external stimuli (e.g., temperature, electromagnetic waves, pH, ions, antigens, antibodies, etc.). This property endows them with great potential for precise drug delivery. Unlike single-stimuli-responsive materials mentioned earlier, these materials can integrate multiple stimuli, further enhancing their "intelligence." Through precise design, they can achieve AND, OR, and even NOT logic operations, integrating multiple stimulus signals to initiate commands. Such materials hold immense potential for the treatment of OM, as materials scientists can design drug release conditions based on the unique microenvironment of OM. This enables precise drug delivery to the localized OM area, reducing drug release in surrounding healthy tissues and achieving targeted therapy. In our literature review, we found no existing studies on using such materials for OM treatment. However, articles exploring the medical potential of these materials do exist. Stauber et al. [147] developed a dual pH- and temperature-responsive nanogel based on sulfoxide-containing poly(methacrylate) (P(nPr-SEMA)-MAA). This nanogel utilizes the thermoresponsive backbone of P(nPr-SEMA), with the addition of MAA to impart pH responsiveness. Upon meeting specific conditions, the gel undergoes swelling, thereby facilitating drug release. Although the study does not directly mention OM treatment, the locally acidic pH and elevated temperature typical of OM infection sites suggest this material holds significant potential for OM therapy.

## 5 Anti-inflammatory biomaterials

Excessive inflammation response is one of the characteristics of OM, which may cause unbearable pain, uncontrolled necrosis of tissue, and even delayed or non-healing [1]. According to the current researches, biomaterials that have antiinflammation ability can reduce inflammation by lowering local ROS level and regulating other inflammatory factors, reducing pain and promoting tissue healing [148]. We will briefly introduce some researches about antiinflammation biomaterials below.

### 5.1 Regulation of ROS

**Fig. 10:**
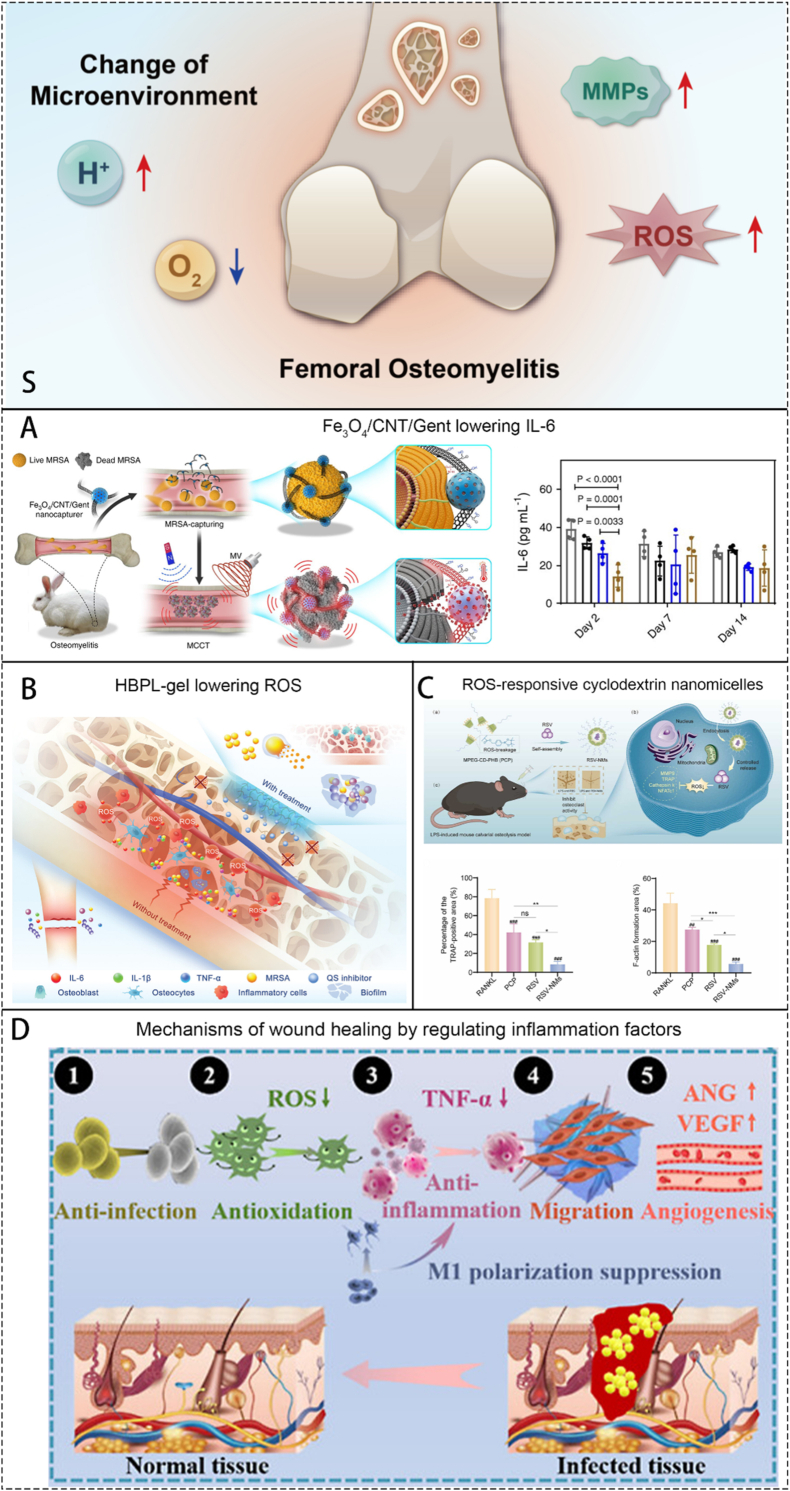
Schematic illustrations of some representative ROS and inflammation factor regulating biomaterials and charts about their excellent effect. S. A scheme of brief introduction of femoral OM environment and the change of several elements. A. The schematic illustration of the microwaveocaloric-chemotherapy (MCCT) of Fe_3_O_4_/CNT/Gent and its effects in lowering IL-6. Reproduced with permission from Ref. [160]. B. Schematic diagram of HBPL-gel-mediated antiinflammation activity by lowering ROS level. Reproduced with permission from Ref. [157]. C. Schematics showing the synthesis of the ROS-responsive resveratrol-loaded cyclodextrin nanomicelles and their application in inhibiting osteoclast formation and their effects in inhibiting the osteoclastogenesis. Reproduced with permission from Ref. [158]. D. Schematics showing the mechanisms of wound healing by regulating inflammation factors. Reproduced with permission from Ref. [161].

Due to the presence of a large number of immune cells, such as phagocytes and lymphocytes, in the inflammatory microenvironment, the cells exert bactericidal effects locally, mainly by releasing enzymes that destroy bacterial cells. During this process, some oxidizing enzymes can cause the local production of a large number of ROS. ROS mainly include O^2−^, H_2_O_2_, hydroxyl radicals, etc. [24] They are also present in small amounts in normal organisms and play an indispensable regulatory role [149]. However, in inflammatory wounds, the presence of excessive reactive oxygen species can lead to local oxidative stress, accelerate local tissue damage, slow wound healing and exert adverse effects on the treatment of OM [150]. Uncontrolled ROS clearance may also cause local ROS levels to be too low, leading to some side effects. Therefore, future research should also focus on sensing the controlled release of drugs at ROS levels to avoid excessive clearance of ROS. Researches on how to lower the level of local ROS to achieve antiinflammation is still ongoing (Fig. 10B). Synthesized and natural substances were both used in current researches. We have developed many kinds of biomaterials with ROS eliminating activity [[151], [152], [153], [154], [155], [156]]. These materials have been used to treat a variety of oxidative damage-related diseases, including atherosclerosis, myocardial infarction, ischemic stroke, and infected wounds. In our further research, we plan to apply these materials to the treatment of OM, an area where related studies are relatively scarce. However, preliminary research in this field has shown that biomaterials with ROS scavenging activity exhibit promising therapeutic effects in the treatment of OM. Zhang et al. [157] focused on the Quorum-Sensing (QS) effect between bacteria cells in post-traumatic OM. They utilized an ROS-responsive bond between N1-(4-borobenzoyl)-N3-(4-borobenzoyl)-the N1, the N1, N3, N3-tetramethylpropane-1,3-diamine (TSPBA) and polyvinyl alcohol (PVA), and the amino side chain of hyperbranched polylysine (HBPL) to lower local ROS level, then inhibit the QS effect, by downregulating key genes of QS, achieving bactericidal effect and promote bone regeneration. Rat tibial OM model proved its effect in killing bacteria, scavenging ROS and inflammation halting. Fang et al. [158] developed a material that balances local ROS levels using polyethylene glycol-based cyclodextrins loaded with resveratrol (RSV) as an antioxidant to neutralize local ROS, to prevent inflammatory osteolysis in OM patients (Fig. 10C). Researchers in our group [159] synthesized a multifunctional dynamic Schiff-base and borate crosslinked glycopeptide hydrogel. Among them, EGCG and PHMS play the role of local clearance of ROS, combined with the antibacterial and angiogenic effects of other materials, and promote local wound healing.

Other biomaterials, instead of lowering ROS level, tried to utilize the high bactericidal effect of it. Many biomaterials were produced to produce high concentration of ROS locally to disrupt the life cycle of bacteria cells. In the Physical therapy part of our review, we mentioned many biomaterials acting as the sensitizers, transforming physical signals into the production of ROS in many ways such as catalyzing. As a result, ROS is a double-sided sword that act in different aspects. In the badly infected OM wounds, elevation of ROS level can accelerate the bactericidal process by recruiting immune cells and directly kill bacteria. In the wounds where infection was well controlled, lowering of ROS can promote the healing process, halt inflammation responses and speed the formation of the bone. So, it is important to judge the infection status of the OM wounds and make the right decision.

### 5.2 Regulation of inflammation factors

In addition of ROS, inflammatory factors such as interleukin (IL), tumor necrosis factor (TNF), interferon (IFN), etc. also play a regulatory role in the local OM inflammatory microenvironment. The effects of pro-inflammatory factors and anti-inflammatory factors are antagonistic to each other, but in the OM wound with severe infection, the pro-inflammatory factors play a dominant role, which enhances the local inflammatory response, destroys cells and delays wound healing. For example, local macrophages type M1 and type M2 secrete factors leading to pro-inflammation and anti-inflammatory respectively.

TNF-α is one of the most focused factors in OM inflammation and healing process. Ma et al. [161] reported a one-component multifunctional bioactivity self-healing scaffolds (itaconic acid - pluronic – itaconic acid) (FIA) to promote MRSA infected wound healing. Itaconic acid exerts antibacterial effects by inhibiting the activity of bacterial isocitrate lyase (ICL). The team analyzed the expression of TNF-α in M1 polarized RAW264.7 cells, and confirmed that FIA can repress the expression of it, thus halt the inflammation overload (Fig. 10D). Both in vivo and in vitro experiments have proved that this material can promote local OM wound healing. Xu et al. [162] focused on the cellular communication. Their copper-based nanoenzyme can regulate Nrf2/NF-κB pathways to interfere with macrophage polarization, thus repress the expression of TNF-α to achieve anti-inflammation effect. Classic bacteria-induced periodontitis model of rats was constructed and proved the osteogenic and antiinflammation effect, which is very promising in utilizing to heal OM wounds. Lowering of TNF-α and rising of TGF-β were observed. In contrast, Li et al. [163] prepared a hydrophilic and viscous hydrogel of poly(vinyl alcohol) modified with chitosan, polydopamine, and NO release donor was formed on a red phosphorus nanofilm deposited on a titanium implant (Ti-RP/PCP/RSNO). As we all know, biofilm formation on OM implants is always an indispensable problem. This material can upregulate the expression of TNF-α by releasing NO, promoting inflammation responses to achieve antifilm effect on OM implants. Implant experiment on rat femur proved its effects on eliminating biofilms. Other factors like IL-6, IL-10 and TGF-β were also made into consideration (Fig. 10A). Qiao et al. [160] found that in the MRSA infected rabbit OM model, inflammatory cells significantly infiltrated the OM infection site by HE staining of marrow cells, including lymph cells, multinucleated giant cells and neutrophils, etc. Inflammation factors were also found with a rising expression. After the treatment of Fe3O4/CNT/Gent nanoparticles, the IL-6 expression was significantly halted at the infection site, WBC count in bloodstream was also lowered, signaling that this material can restrict the inflammatory reaction of OM rabbit both locally and systematically. Shen et al. [164] and Jin et al. [165] both mentioned the factors above as indicators of inflammation response. Shen's material lowered the expression of TNF-α, IL-6 and IL-10, Jin's material lowered IL-6 but elevated IL-10.

## 6 Immunoregulatory biomaterials

At the OM infection site, various immune cells gather. Precursor immune cells come first, such as granulocytes and macrophages. They break up and release antigens of the pathogens. Then T cells and B cells are also our focus. Both of them play their part in killing, targeting, helping, etc. In total, regulating the activity of local immune cells to relieve local inflammatory response and promote wound healing is a thought worthy of further exploration. Various researchers mentioned the concept of immunoregulatory effect in OM anti-inflammatory adaptations. Chu et al. [166] mentioned the concept of Foreign Body Response (FBR). This concept holds that foreign implants can have a series of interactions with local immune cells, thereby affecting local immune regulatory processes represented by macrophages and cytokine regulatory processes. Xu et al. [167] also mentioned the biomaterial-related immune regulation in accelerating the healing of chronic wounds, mainly related to the change between M1 and M2 macrophages. Chen et al. [168] summarized the main ways to utilize biomaterials to control immune cells, including coating, adding external substances, adjusting the surface, change the diameter of the fiber, porosity, etc. Most researches focus on adding external substances because of the simplicity and proved effectivity.

**Fig. 11:**
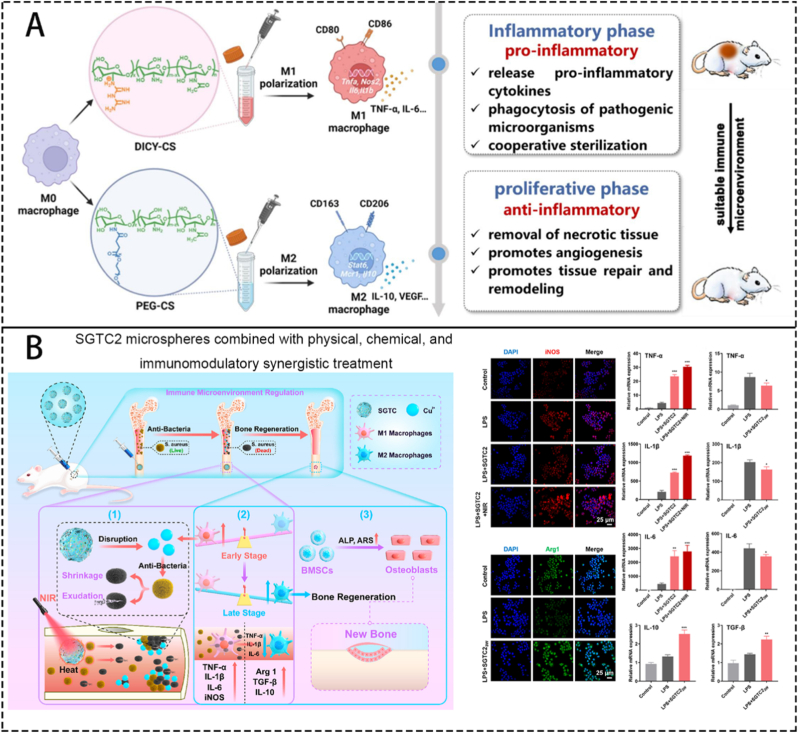
Schematics, photos and charts showing the use of macrophage polarization-regulating biomaterials and their excellent healing ability. A. Brief schematics showing specific cell targets in the process of macrophage polarization and the special work of M1 and M2 in the non-specific immune process. Reproduced with permission from Ref. [174]. B. (Left) Diagram of the mechanisms utilizing SGTC2 microspheres combined with physical, chemical, and immunomodulatory synergistic treatment of Acute OM and (Right) representative immunofluorescence images of the surface biomarkers of M1 and M2 macrophage and related gene expression levels. Reproduced with permission from Ref. [164].

Macrophages undergo the process of polarization, becoming M1 and M2 macrophages, to promote 2 different responses of healing process. M1 promotes inflammatory responses, upregulates the secretion of pro-inflammatory cytokines to achieve bactericidal effects. While M2 downregulates inflammation and promotes the growth of new tissue. In OM wounds, M1 upregulates osteoclasts and M2 promotes osteoblasts, the ratio between M1 and M2 significantly affects the osteogenic activity of local OM wounds (Fig. 11A). Here are various ways of changing the ratio between M1 and M2 macrophages. One is to add drugs to interfere with the biological process of macrophage polarization. Many kinds of natural and synthesized substances were proved having this effect. Shen et al. [164] developed injectable microspheres composed of an interpenetrating network of ionically cross-linked sodium alginate (SA) and genipin (Gp)-cross-linked gelatin (Gel), loaded with tannic acid (TA) and copper ions (Cu^2+^) as PTT agents. *In vitro* cell culture results demonstrated that these microspheres can adaptively and sequentially modulate macrophage polarization between the M1 and M2 phenotypes. The SA network, formed through calcium ion cross-linking, provides structural integrity to the microspheres, while the Gp–Gel cross-linking establishes a second network, creating a natural photothermal conversion system. During the initial treatment phase, the microspheres release high concentrations of Cu^2+^, which, combined with PTT, promotes M1 macrophage polarization for early anti-infection effects. In later stages, reduced Cu^2+^ release and increased TA release shift macrophage polarization toward the M2 phenotype, facilitating bone repair. The therapeutic efficacy was validated in a Sprague–Dawley (SD) rat model of MRSA-induced OM in the right femur, which confirmed excellent PTT performance, bacterial clearance, new bone formation, and elevated expression of the M2-specific biomarker Arg1. (Fig. 11B). Chen et al. [169] developed Chitosan@Puerarin(C@P). This material has good biocompatibility and can promote the healing of uninfected wounds by inhibiting the formation of M1 and promoting the formation of M2. Deng et al. in our team [170] prepared a wound dressing using phenylboronicacid grafted ϵ-polylysine (EPBA) and poly-vinylalcohol (PVA) combined with astragaloside (AST), which can play a role in local regulation of macrophage polarization. Another method is to introduce alien cells instead of local cells to induce polarization. Yu et al. [171] designed an embedding system with bone marrow derived macrophages (BMMs) and also contains BMSCs, using different materials. In the rat skull defect model, BMSCs can promote the polarization of BMMs to M2, and thus promote bone healing. Considering about its great osteogenic effects, this material can be a very promising material in OM curing while promoting osteogenesis. The third method is to design specific surfaces combining with driving factors. As we know, OM surgical treatment widely includes Ti implants, but implant-associated infection may result in implant failure. So, it is very important to prevent such infection in OM treatment. Li et al. [172] made a metal-piezoelectric hetero-nanostructure with mechanical energy-driven antimicrobial property to prevent surface infection on titanium implants. Under ultrasonic irradiation, macrophages display potent phagocytosis and anti-bacterial activity through the activation of PI3K-AKT and MAPK pathway. RAW264.7 cells polarization experiment were carried and proved its M1 promoting activity under US irridation. Implant associated OM model were made with male rats and proved its antiinflammation effect by H&E staining. Xie et al. [173] induced a bilayered hydrogel contaning different drugs to promote antibacterial effect in OM patients. In vitro test proved the hydrogel itself have the effect of promoting M1 polarization locally.

**Fig. 12:**
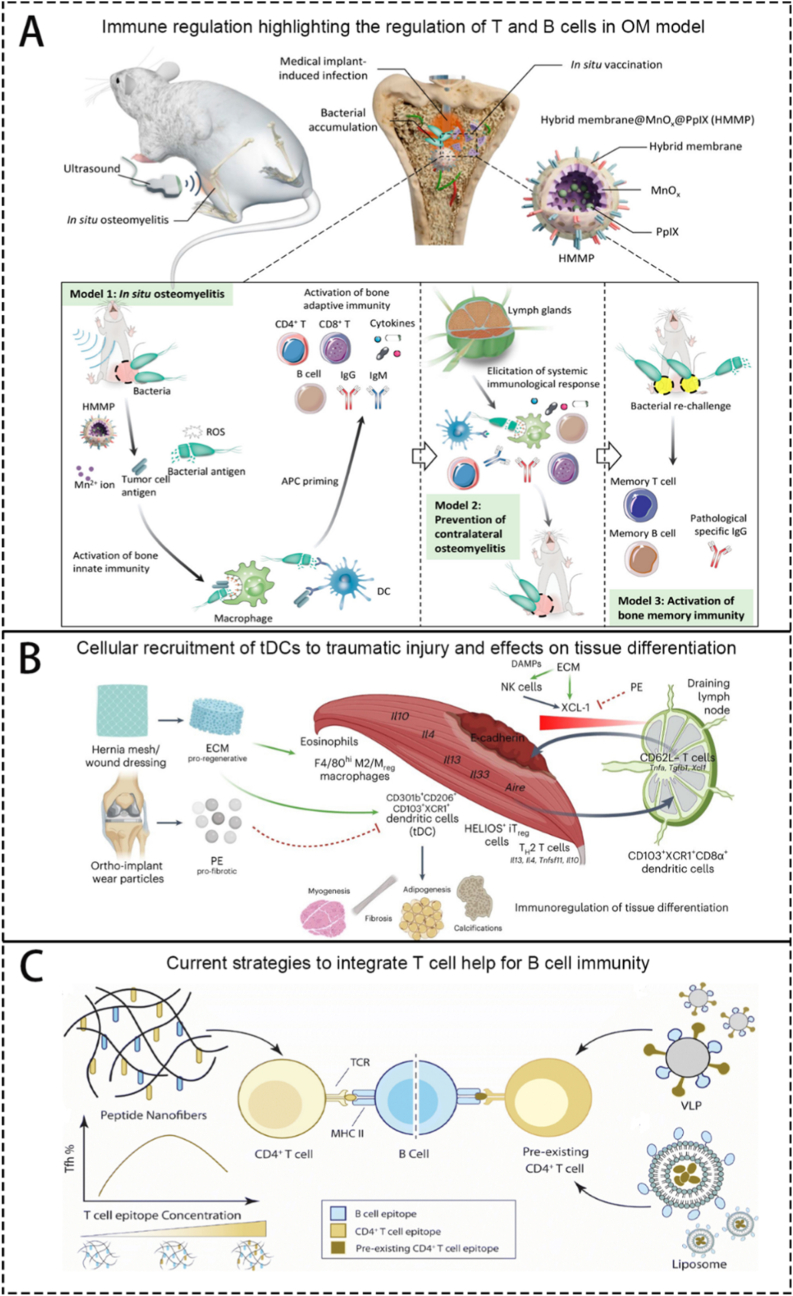
Schematics showing the mechanisms of T/B cell regulation and some utilization of representative T/B cell regulatory biomaterials. A. Schematics of immune regulation highlighting the regulation of T cells and B cells in OM model. Reproduced with permission from Ref. [175]. B. Schematic of cellular recruitment of tDCs to traumatic injury and subsequent effects on tissue differentiation. Reproduced with permission from Ref. [177]. C. Schematics showing the current strategies to integrate T cell help for B cell immunity. Reproduced with permission from Ref. [176].

Cytotoxic T cells play a significant role in the formation of chronic OM. These cells are depleted under the stimulation of a large number of inflammatory factors, leading to dysfunction, which inhibits the secretion of killing substances like perforin, serine esterase and interferon, etc., upregulates inhibitory substances like TGF-β, IL-4 and IL-10, etc., and promotes the chronicity of OM [25]. B cells secrete specific antibodies to mark the target bacteria antigen, accelerating the bactericidal process. Regulating local antigen presentation processes and humoral immunity (the activity of B cells) to promote tissue growth and healing is the most popular way of immunoregulation. But there is little research on the use of this method to treat OM. Lin et al. [175] induced the concept of in-situ vaccination, and made a biomimetic nanomedicine that can trigger local immune response, activate both T and B cells (Fig. 12A). It was made with HMMP, which was constructed by engineering PpIX-encapsulated hollow MnO_x_ with a hybrid membrane exfoliated from both macrophage and tumor cell lines. Encapsulating bacterial antigen with HMMP, inject it into the OM infection site, and release it by SDT. In rat OM model, this vaccine performed positive effects in controlling OM while significantly prevented the recurrence of OM. Splenocytes collected from mice immunized with HMMP exhibit the highest infiltration of CD4^+^ and CD8^+^ T cells as well as NK cells, inhibited the influx of regulatory immune cells including MDSCs, thus reducing the immunosuppressive potential of bacterial infection. Then providing specific immunity against OM infections. Other researches mainly focused on cancer treatment. Zareein et al. [176] discussed in their study about the biomaterial transformation method that can interfere with humeral immunity and B cell behavior, and the use of bio-engineering B cells in the preparation of the vaccine that can interfere with immune systems (Fig. 12C). Lokwani et al. [177] found the biomaterials can promote BATF3 dependent dendritic cell proliferation by studying the commonly used biomaterials in the process of muscle tissue healing effect on the immune cells, affects the processing of antigen (Fig. 12B). Both of the materials have the potential to be used to cure OM due to their proven healing effects.

Although there is a lack of specific studies on immunomodulatory treatment of OM, we speculate that the immunomodulatory mechanisms mentioned above may play a positive role in the treatment of OM. For example, the more-speculated polarization aspect of macrophages. By maintaining the balance of M1 and M2 macrophages, local inflammation and repair response can be balanced, so that antibacterial and repairing process are maintained in a delicate balance. Thus, it can promote angiogenesis and new bone formation in the inflammatory site of bone marrow. If there are too many M1 macrophages, local inflammation is too strong and is not conducive to repair, and too many M2 may lead to scar tissue formation. Neither leads to the recovery of OM [168]. The effect of antigen presentation process is complex, involving multiple stages, but the final effect is mainly focused on promoting tissue growth and inhibiting local inflammatory response [178].

## 7 Tissue-regeneration promoting biomaterials

Despite the substantialness of the researches on biomaterials that promote tissue regenerating process, current studies on biomaterials for treating OM have not prominently emphasized the importance of the 2 characteristics: angiogenesis and osteogenesis. Here, we emphasize and summarize biomaterials that exhibit the aforementioned characteristics in the treatment of OM, aiming to fill the gap in this field.

### 7.1 Angiogenic biomaterials

Angiogenesis is essential in the treatment of OM. In the OM wounds, due to the stimulation of inflammatory factors and change of the local microenvironment, angiogenesis effect is inhibited, resulting in new blood vessels cannot easily distribute into the infected site. Local blood supply is reduced, nutrients, drugs and immune cells cannot be easily distributed, and bacterial metabolites and cell debris cannot be removed. These phenomena significantly inhibit local wound healing and increase the risk of chronic inflammation. In the course of treating OM, even in the course of the treating most orthopedic diseases, promoting local angiogenesis is a necessary step. In our recent researches [[179], [180], [181], [182], [183], [184]], we have also developed several biomaterials with excellent angiogenic activity. These materials can effectively promote vascular regeneration in ischemic diseases by enhancing the secretion of multiple growth factors. Although we have not yet applied these materials to the treatment of OM, we believe that similar materials with angiogenic properties could be well-suited to meet the treatment needs of OM and have significant potential for application in this field.

**Fig. 13:**
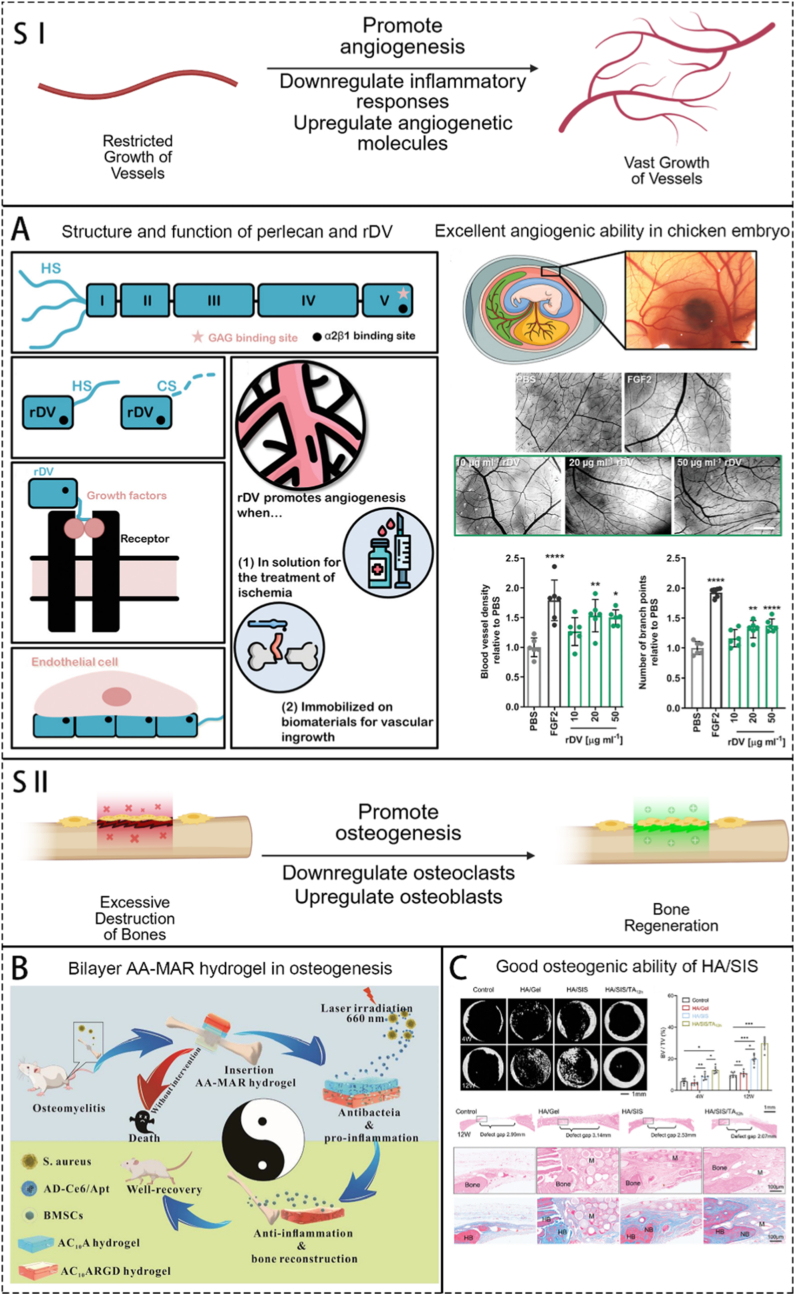
Schematics of representative angiogenesis and osteogenesis-related biomaterials and photos and charts showing their excellent healing ability. S I: A scheme of brief introduction of angiogenesis mechanisms. A. (Left) Schematic illustration of the structure and function of perlecan and recombinantly expressed C-terminal domain V region (rDV) and (Right) its excellent angiogenic ability in chicken embryo. Reproduced with permission from Ref. [185]. S II: A scheme of brief introduction of osteogenic mechanisms. B. Schematic illustration of the bilayer AA-MAR hydrogel for spatiotemporal modulation of bactericidal and osyeogenic process in OM treatment. Reproduced with permission from Ref. [173]. C. Micro-CT image and staining images showing good osteogenic ability of the HA/SIS-Based composite scaffolds. Reproduced with permission from Ref. [189].

There is a considerable amount of review on biomaterials that promote local angiogenesis. Some researches focused on constructing new surfaces or domains on specific biomaterials to promote vascularization. Lin et al. [185] studied recombinantly expressed domain V(rDV) as a way of enhancing growth factor signaling, thus promote angiogenesis on implanted biomaterials. rDV enhances endogenous growth factor signaling through its glycosaminoglycan chain, thereby promoting angiogenesis. They fixed the rDV onto a three-dimensional porous fibroin biomaterial, promoting the growth of blood vessels and the fusion of the implanted scaffold with the surrounding tissue. In vivo test proved that rDV can promote the signal molecule FGF2 in chicken embryo to upregulate, thus promoting angiogenesis. Subcutaneous implantation in mice wound model also proved its good vascularizing ability (Fig. 13A). Implanted materials are widely used in the curing surgery of OM. We believe that this material can also be used in treating OM by making enough modifications. Adding new biomaterials onto the implanted materials can significantly accelerate the curing process of OM wounds. Xia et al. [178] studied the adjusting function of mesoporous silica (MS) towards the adaptive immune T cells in the promotion of local blood vessels and bone formation. MS extract can inhibit the expression of regulatory factor X-1(RFX-1) in CD4^+^T lymphocytes, and the supernatant of CD4^+^T lymphocytes preconditioned with MS extract can significantly promote the regeneration of vascularized bone. 6-week-old mice model with skull defect were used to investigate the angiogenic effect, and CD31, Ang-1, VEGF, and von Willebrand Factor (vWF), were significantly upregulated in the skull defects of the MS group compared to the control group, indicating its great angiogenic effect in bone defects, which is very useful in OM healing. Adding biological substances to regulate cell behavior is also big research topic. Sadowska et al. [186] constructed a multifunctional collagen-based scaffold containing microRNA-138 inhibitor (antagomiR-138). Rat femoral bone defect model was utilized to perform in vivo experiment, and proved its reconstruction effect in bone healing. Our team focused on utilizing natural substances to promote angiogenesis. One research [187] made hydrogel containing paeoniflorin for chronic diabetic wound. The release of paeoniflorin was achieved through the response to ROS, resulting in the role of promoting angiogenesis. Another [188] utilized hydrogel with similar construction, incorporated magniferin to achieve angiogenesis. Both obtained good vascularization in rat model.

### 7.2 Osteogenic biomaterials

Local bone destruction and generation in OM are directly related to the prognosis of OM. As mentioned earlier, *S. aureus* infection can trigger an imbalance between M1 and M2 macrophages [26]. These two types of cells indirectly regulate osteogenic and osteoclast behavior by regulating the activity of osteoblasts and osteoclasts. In this imbalanced situation, the activity of osteoclasts dominates, leading to increased local bone destruction in inflammation, expansion of bone cavities, and spread of infection. The inflammatory microenvironment formed locally in OM also plays an indispensable role in the progression and maintenance of OM. Delayed bone healing is a very big problem in the current treatment of OM, which can lead to bone defects and even nonunion, then provides a good environment for bacterial reproduction and immune escape, leads to chronic manifestations such as sinus formation. Promoting osteogenesis can accelerate the healing of local bone defects, prevent OM from becoming chronic, and improve the cure rate.

There is a very considerable amount of researches mentioned osteogenesis effect. A wide range of different ways are also mentioned. The most utilized way is to *incorporate.* Anything that can promote osteogenesis can be made to be incorporated onto biomaterials to apply locally. Some of them directly promotes osteogenesis. Wang et al. [46] induced N-halamine onto the surface porous PEEK, which exhibits wide-range antibacterial, antifungal, anti-inflammatory, and osteogenic effects. When N-halamine is consumpted, it can turn into a new substance that has even better osteogenic effect. Rat implant-associated OM model confirms that sp-PEEK-NCl reduces bone destruction caused by infection and promotes osteointegration in vivo. Inflammatory factors like TNF-α and iNOS were significantly downregulated in contrast with single PEEK implants, and significant bone regeneration was observed under micro CT. Song et al. [189] developed a composite scaffold with excellent cytocompatibility and facilitates superior bone regeneration. It integrates a self-assembled small intestinal submucosa (SIS) hydrogel with pre-adsorbed HAP particles and tannic acid (TA), showing special mechanical resilience and porous structures, suitable for filling into amorphous cavities and promoting cell infiltration. Rat calvarial defects with *S. aureus* infection model were constructed and proved the osteogenic effect of this composite. It successfully eradicated local *S.aureus* infection, and elevated local VEGF and TGF-β secretion, thus promoted bone healing. This effect gives it a great potential in healing OM. Xie et al. [173] prepared a bilayer hydrogel based on genetically engineered polypeptide AC_10_A and AC_10_ARGD, named as AA-MAR. The upper layer contains Ag_2_S QDs@DSPE-mPEG_2000_-Ce6/Aptamer (AD-Ce6/Apt) for antibacterial purposes, while BMSCs were incorporated in the lower second layer to promote osteogenesis. *S.aureus* induced OM model of rats was constructed and functional fracture healing at 5 weeks post operation was evaluated. The result showed AA-MAR with laser irridation significantly promoted bone healing by testing bone volume, 3-point bending, maximum loading and stuffness (Fig. 13B).

Our team members [190] utilized carboxymethyl chitosan (CCS), oxidized hyaluronic acid (OHA) and tannic acid (Ta) to prepare an injectable self-healing hydrogel (Ta@gel). And the bone marrow mesenchymal stem cells (BMSCs) were wrapped in microspheres composed of GelMA. The microspheres are loaded into Ta@gel. This combination forms a biomimetic three-layer composite structure that resembles the natural osteochondral complex. Among them, BMSCs showed considerable bone regeneration ability. New Zealand rabbit osteochondral defect model was used and proved the chondrogenic effect. In addition, PLL-incorporated HAP scaffolds were observed to have better osteogenic activity by enhancing the osteogenic differentiation of incorporated BMSCs. Other researches focused on regulating polarization of M1 and M2 macrophages to promote osteogenesis, in which we mentioned in the immunomodulation part. In addition [191,192], we have utilized surface modification to coat magnesium and titanium alloy medical implants with cationic quaternary ammonium salts (QAs) or Mg-EGCG metal-polyphenol networks, which have osteogenic properties. These surface-modified implants exhibit good antibacterial and anti-inflammatory activities and can effectively promote bone integration.

The functions of the biomaterials mentioned above mainly focus on anti-inflammatory effects, immune regulation, and promoting tissue growth. Unlike conventional antibacterial biomaterials, which primarily concentrate on antimicrobial properties, these materials emphasize facilitating wound healing and bone growth. They are generally used after local sterilization is completed and infection is under control. The comparisons between these biomaterials are summarized in Table 3 below.

**Table 3:** Comparisons between various kinds of anti-inflammatory, immunomodulatory and tissue-regeneration promoting biomaterials.

Antiinflammatory/Tissue regenerating agent	Mechanism	Anti-bacterial activity	Angio-genic activity	Osteo-genic activity	Carrier/Form	Ref.
Inflammatory Microenvironment-Responsive Hydrogels with QS-inhibitor.	Scavenge local ROS to produce a Quorum-sensing-free environment.	Yes	Not mentioned	Yes	Hydrogel	157
ROS-responsive resveratrol-loaded cyclodextrin nanomicelles	Resveratrol scavenges local ROS to enhance osteogenic.	Not mentioned	Not mentioned	Yes	Nanomaterials	158
Poly(itaconic acid-pluronic)(FIA) hydrogel	FIA produce robust antibacterial antioxidant, lowering the expression of TNF-α	Yes	Yes	Not mentioned	Hydrogel	161
Microenvironment-Responsive Metal-Phenolic Nanozyme Release Platform	CuTA nanozyme local release, scavenge ROS and lowering expression of TNF-α	Yes	Not mentioned	Yes	Nanomaterials	162
Ti-RP/PCP/RSNO	Release NO under infrared light locally, upregulates the expression of TNF-α	Yes	Not mentioned	Yes	Hydrogel	163
MW-responsive Fe_3_O_4_/CNT and gentamicin	precise bacteria-capturing ability and magnetic targeting, release GEN and heat locally.	Yes	Not mentioned	Not mentioned	Nanomaterials	160
Multifunctional Injectable Microspheres Containing “Naturally-Derived” Photothermal Transducer	Sequential Immunomodulation interfering the polarization of macrophages.	Yes	Not mentioned	Yes	Nanomaterials	164
Mechanical Force Induced Self-Assembly of Chinese Herbal Hydrogel	Inhibiting the formation of M1 and promoting the formation of M2	Yes	Yes	Not mentioned	Hydrogel	169
Biomimetic Nanomedicine-Triggered *in Situ* Vaccination (HMMP)	A biomimetic nanomedicine that can trigger local immune response, activate both T and B cells.	Not mentioned	Not mentioned	Not mentioned	Nanomaterials	175
Bone Marrow-Derived Macrophages Combined with Bone Mesenchymal Stem Cells in scaffolds	BMSCs can promote the polarization of BMMs to M2	Not mentioned	Not mentioned	Yes	Scaffold modifications	171
A metal-piezoelectric hetero-nanostructure with mechanical energy-driven antimicrobial property	Produce ROS and promote the polarization to M1 phages, halt the formation of biofilms.	Yes	Not mentioned	Not mentioned	Scaffold modifications	172
Self-assembled bilayer polypeptide-engineered hydrogel	Different drugs in different layers producing antibacterial and M1 promoting action.	Yes	Not mentioned	Yes	Hydrogel	173
Recombinantly Expressed Domain V of Human Perlecan (rDV)	3D porous silk fibroin biomaterials integrated with rDV to promote angiogenesis.	Not mentioned	Yes	Not mentioned	Scaffold modifications	185
Silicon-Based Biomaterials: Meosporous silica (MS)	MS extract reduce the expression of RFX-1 in CD4^+^ Tc, enhances angiogenesis and osteogenesis.	Not mentioned	Yes	Yes	Nanomaterials	178
Microenvironment-responsive hydrogel	Nano-ZnO for killing microbes. Paeoniflorin-encapsulated micelle for angiogenic activity	Yes	Yes	Not mentioned	Hydrogel	187
A spatiotemporal release platform based on pH/ROS stimuli-responsive hydrogel	Magniferin promotes angiogenesis, diclofenac sodium produces anti-inflammatory effects.	Yes	Yes	Not mentioned	Hydrogel	188
Polyetheretherketone with a Porous *N*-halamine polymeric coating	N─Cl groups convert into N─H groups, promote antibacterial and osteogenic effects.	Yes	Not mentioned	Yes	Polymers Scaffold coatings	46
Mechanics-resilient HA/SIS-based composite scaffolds	HAp particles and tannic acid were pre-absorbed to promote ROS scavanging and osteogenesis.	Yes	Not mentioned	Yes	Bioceramics	189
Customized triphasic cartilage composite scaffold	CS, OHA, and Ta were used to create an anoxic microenvironment within the hydrogel.	Not mentioned	Not mentioned	Yes	Hydrogel	190
A Multifunctional Scaffold with microRNA therapy and antimicrobial nanoparticles	Copper-doped bioactive glass and inhibitor of microRNA-138 to stimulate antibacterial and regeneration process.	Yes	Yes	Yes	Nanomaterials	186

## 8 Multifunctional biomaterials

As summarized earlier, we have categorized representative biomaterials based on specific functionalities. However, biomaterials integrating multiple functions have also become a major research focus. These materials incorporate diverse mechanisms, potentially carry multiple drugs, and can perform simultaneous actions such as antibacterial activity, osteogenesis promotion, and absorption of local necrotic tissue. In a recent study in 2025, Yang et al. [193] developed a multifunctional biocapsule with both antibacterial and immunomodulatory effects. Constructed via Schiff base reactions of procyanidins, the capsule is co-loaded with the osteogenic peptide K6-OGP and tobramycin to achieve dual antibacterial and osteogenic functions. The team also found that the biocapsule modulates the OM microenvironment by catalyzing ROS decomposition and promoting M2 macrophage polarization, thereby steering OM toward recovery. The material demonstrated significant anti-Staphylococcus aureus effects, and its angiogenic and osteogenic capabilities were well validated in a rat OM model. Similarly, Shou et al. [194] designed self-assembled curcumin-Sr metal-phenolic network nanoparticles, which were surface-modified with a hexameric lysine-conjugated antimicrobial peptide (K6-CAMEL0). The system exhibited broad-spectrum antibacterial activity, along with ROS scavenging, pro-angiogenic, necrotic bone clearance, and osteogenic effects. In animal models, it successfully eradicated *S. aureus* biofilms and promoted local bone regeneration. Additionally, Lu et al. [195] integrated calcium phosphate (CaP) with GelMA microspheres co-loaded with oligonucleotides (Oligo) and vancomycin (Van), creating an injectable nanomaterial termed CaP@MS-Oligo-Van. This system provides combined anti-inflammatory, antibacterial, and osteogenic functions. In recent years, studies of this kind have been emerging frequently, reflecting a clear trend in OM therapeutic strategies toward multifunctional biomaterials. This progress is enriching the possibilities for tailored OM treatments.

## 9 Conclusion and outlook

Current OM treatment involves high-dose systematic antibiotics and local surgical debridement, but due to the diverse classification of OM and the personalized differences in treatment needs, the therapeutic outcomes are often unsatisfactory. Recently, biomaterials have brought new hope for OM therapy. Researchers have endowed the biomaterials with various functions through chemical modification or incorporation. However, current research is scattered, with most biomaterials limited to 1 or 2 functions, hindering their application in complex clinical scenarios. Thus, summarizing and categorizing these functional materials is essential to offer new perspectives for biomaterial construction and expedite their clinical translation for OM treatment. This review integrated the OM microenvironment and the principles of constructing multifunctional biomaterials and their therapeutic mechanisms, then further explored the personalized customization of multifunctional biomaterials, aiming to broaden research perspectives and meet complex clinical demands. Functional biomaterials for OM treatment remain in the early stages of development, and researches face several challenges and issues to be addressed.

i) Curing OM is challenging. Thus, developing drug delivery systems for sustained release of antibacterial substances or synthesizing bioactive materials that activate adaptive immunity is crucial to prevent OM recurrence.
ii) While enhancing the antibacterial activity of biomaterials can increase cytotoxicity, Future research should focus on developing biomaterials with both good biocompatibility and selectivity, such as antimicrobial peptides and engineered bacteriophages.
iii) Appropriate biodegradability is crucial for biomaterials. Thorough assessment of their biosafety, biocompatibility, and biodegradability is necessary. Additionally, since OM often involves bone destruction, biomaterials with degradation rates matching bone regeneration are more beneficial for clinical treatment.
iv) Smart stimulus-responsive biomaterials offer unique advantages for the complex microenvironment of OM. These materials can break down in response to changes in pH, ROS, MMPs, temperature, etc., delivering bioactive substances in a controlled manner for precise treatment. Additionally, biosensing biomaterials can monitor local microenvironment changes during treatment, enabling timely adjustments in therapeutic strategies.
v) Since biomaterials for treating OM are still in the preliminary stage, their specific mechanisms are unclear. Future research should focus on treatment mechanisms, such as antibiotic resistance, pathogenic bacteria immune evasion, adaptive bacterial immunity, recruitment of immune and inflammatory cells, and bone metabolic balance.
vi) Given the diverse classifications and causes of OM in clinical settings, future biomaterial designs should consider the varying treatment needs of different OM types, particularly those complicated by diabetes or other underlying conditions. It is essential to develop customized multifunctional bioactive materials tailored to target specific pathogens and adapt to various forms of OM.

In summary, developing multifunctional biomaterials offers new therapeutic strategies for OM with promising clinical translation potential. By summarizing and analyzing recent literature, we aim to provide a comprehensive perspective to drive the design, development, and application of these materials for OM treatment. Despite remaining challenges before clinical adaptation, intelligent multifunctional biomaterials hold significant clinical promise for future OM patients.

## CRediT authorship contribution statement

**Qi-Rui Geng:** Writing – review & editing, Writing – original draft, Investigation, Conceptualization. **Ya-Xing Li:** Writing – review & editing, Data curation, Conceptualization. **Heng Gong:** Writing – review & editing, Conceptualization. **Ting-Jiang Gan:** Writing – review & editing, Conceptualization. **Shi-Jiu Yin:** Writing – review & editing, Conceptualization. **Xi-Kun Ma:** Writing – review & editing, Conceptualization. **Cheng Zheng:** Writing – review & editing, Conceptualization. **Ye Wu:** Writing – review & editing, Writing – original draft, Supervision, Software, Resources, Methodology, Funding acquisition, Data curation, Conceptualization. **Hui Zhang:** Writing – review & editing, Supervision, Methodology, Investigation, Funding acquisition, Data curation, Conceptualization. **Yun-Bing Wang:** Writing – review & editing, Supervision, Resources, Conceptualization.

## Declaration of competing interest

The authors declare that they have no known competing financial interests or personal relationships that could have appeared to influence the work reported in this paper.

## Data Availability

No data was used for the research described in the article.
